# Outcomes of Systemic Treatment in Children and Adults With Netherton Syndrome: A Systematic Review

**DOI:** 10.3389/fimmu.2022.864449

**Published:** 2022-03-30

**Authors:** Anouk E. M. Nouwen, Renske Schappin, N. Tan Nguyen, Aviël Ragamin, Anette Bygum, Christine Bodemer, Virgil A. S. H. Dalm, Suzanne G. M. A. Pasmans

**Affiliations:** ^1^Department of Dermatology-Center of Pediatric Dermatology, Erasmus University Medical Center-Sophia Children’s Hospital, Rotterdam, Netherlands; ^2^Department of Clinical Genetics, Odense University Hospital, Odense, Denmark; ^3^Clinical Institute, University of Southern Denmark, Odense, Denmark; ^4^Department of Dermatology, Reference Centre for Genodermatoses and Rare Skin Diseases (MAGEC), Necker-Enfants Malades Hospital (AP-HP), Paris Centre University, Paris, France; ^5^Department of Immunology, Erasmus University Medical Center, Rotterdam, Netherlands; ^6^ Department of Internal Medicine, Division of Allergy & Clinical Immunology, Erasmus University Medical Center, Rotterdam, Netherlands

**Keywords:** systematic review, Netherton syndrome, Ichthyosis linearis circumflexa, *SPINK5*, erythroderma, skin disease, ichthyosis, therapy

## Abstract

**Background:**

Comèl-Netherton syndrome (NS) is a rare disease caused by pathogenic variants in the *SPINK5* gene, leading to severe skin barrier impairment and proinflammatory upregulation. Given the severity of the disease, treatment of NS is challenging. Current treatment regimens are mainly topical and supportive. Although novel systemic treatment options for NS have been suggested in recent literature, little is known about their outcomes.

**Objective:**

to provide an overview of systemic treatment options and their outcomes in adults and children with NS.

**Methods:**

Embase, MEDLINE, Web of Science, Cochrane Central Register of Controlled Trials, and Google Scholar were searched up to July 22, 2021. Empirical studies published in English language mentioning systemic treatment in NS were enrolled. Studies that did not define a treatment period or report at least one outcome were excluded. Methodological quality was evaluated by the Joanna Briggs Institute critical appraisal checklist for case reports or case series. Overall quality of evidence of the primary outcome, skin, was assessed by the GRADE approach.

**Results:**

36 case series and case reports were included. The effects of 15 systemic therapies were described in 48 patients, of which 27 were children. Therapies included retinoids, prednisolone, cyclosporine, immunoglobulins, and biologicals. In retinoids both worsening (4/15 cases) and improvement (6/15 cases) of the skin was observed. Use of prednisolone and cyclosporine was only reported in one patient. Immunoglobulins (13/15 cases) and biologicals (18/21 cases) showed improvement of the skin. Certainty of evidence was rated as very low.

**Conclusion:**

NS is a rare disease, which is reflected in the scarce literature on systemic treatment outcomes in children and adults with NS. Studies showed large heterogeneity in outcome measures. Adverse events were scarcely reported. Long-term outcomes were reported in a minority of cases. Nonetheless, a general beneficial effect of systemic treatment was found. Immunoglobulins and biologicals showed the most promising results and should be further explored. Future research should focus on determining a core outcome set and measurement instruments for NS to improve quality of research.

**Systematic Review Registration:**

https://www.crd.york.ac.uk/prospero/display_record.php?RecordID=217933, PROSPERO (ID: 217933).

## 1 Introduction

Comèl-Netherton syndrome (NS; OMIM 256500) is a rare and severe, potential life-threatening disorder of epidermal maturation and keratinization (1). It has an incidence of 1 per 200.000 births and an estimated prevalence of 1-9/1.000.000 (2). It is caused by pathogenic autosomal recessive homozygous or compound heterozygous variants in the serine protease inhibitor of the Kazal type 5 (*SPINK5*) gene on chromosome 5q32 (1, 3, 4). To date, over 80 different variants have been reported (4). The *SPINK5* gene normally encodes LEKTI (lympho-epithelial Kazal-type-related inhibitor), an epidermal serine protease inhibitor that is crucial for maintaining skin barrier function (5–7). The result of pathogenic variants in *SPINK5* is a continuous break-down of the skin barrier with secondary inflammation. Although *SPINK5* is also expressed in other tissues including esophagus, tongue, and thymus, the consequences of the pathogenic variants in *SPINK5* in these tissues remain unclear (7, 8).

LEKTI deficiency in the granular layer of the epidermis and in the inner root sheets of hair follicles leads to a loss of inhibition of serine proteinases and unopposed activity of kallikrein-related peptidase 5 (KLK5), which activates KLK7, KLK14, and elastase 2 (ELA2) (5, 9–14). The deregulated activity of KLKs leads to an increased degradation of desmosomal cadherin component desmoglein 1 (DSG1) and other corneodesmosomal proteins resulting in loss of cell adhesion, desquamation, and early detachment of the stratum corneum (SC) (15–17). This causes severe skin barrier impairment. Through proteinase-activated receptor 2 (PAR2) signaling in keratinocytes, KLK5 triggers the secretion of proinflammatory and proallergic cytokines, including tumor necrosis factor-a (TNF-a), Intercellular Adhesion Molecule 1 (ICAM-1), Interleukin-8 (IL-8), and thymic stromal lymphopoietine (TSLP) (18–20). This further enhances allergic predisposition and secondary inflammation (21). Furthermore, a recent study in patients with NS showed striking upregulation of the T helper type 17 (Th17)/IL-23 pathway and IL-1β expression, suggesting a potential target for systemic treatment (22).

NS is clinically characterized by congenital ichthyosiform erythroderma, trichorrhexis invaginata (bamboo hair), and atopic manifestations (food allergy, asthma, rhinoconjunctivitis) with high IgE levels and eosinophilia (23–25). Especially in the first year, patients have a high risk of life-threatening complications, including hypernatriemic dehydration due to extensive transepidermal waterloss, hypothermia, failure to thrive, and sepsis (1, 26–29). In a majority of patients, the erythroderma present at birth gradually evolves into ichthyosis linearis circumflexa with typical ‘double-edged’ scales (30). Although symptoms can vary widely, most patients experience extensive pruritus and recurrent skin infections (24, 29, 31). Furthermore, NS is considered an immunodeficiency, based on previous studies that showed defects in immune cell function (29, 32, 33). However, a recent study suggests a local skin barrier defect in the absence of an underlying systemic immunodeficiency (24).

Given the severity of the disease, treatment of NS is important but often challenging. Little is known about therapeutic options and their outcomes, with treatment regimens hitherto being only supportive in nature. Furthermore, current treatment strategies mainly target skin symptoms. Although lifelong treatment is required, there are no registered treatments and treatment guidelines available for patients with NS. First-step treatment options include topical corticosteroids (34). Also topical calcineurin inhibitors and narrowband ultraviolet B phototherapy have been reported in NS patients (35, 36). For many patients systemic agents are required. Retinoids have been described in several studies (37–39). In recent years, novel systemic treatment options for NS have been suggested in the literature, such as treatment with immunoglobulins, and biologicals targeting specific inflammatory pathways (29, 40, 41).

A recent systematic review summarized the different therapeutic options for children with NS (42). Here, we further explore treatment regimens for NS, specifically focusing on systemic treatment options in both children and adults. This systematic review aims to give an overview of systemic treatment options and their outcomes in patients with NS as a basis for future guideline development.

## 2 Methods

### 2.1 Search Strategy

A comprehensive systematic literature search was performed in Embase, MEDLINE, Web of Science, Cochrane Central Register of Controlled Trials, and Google Scholar up to July 22, 2021.We performed a broad search for all articles including NS patients. For the full search strategy see **Supplementary 1**.

### 2.2 Eligibility Criteria/In- and Exclusion Criteria

A pragmatic approach was used to include as many studies as possible for this systematic review. We included all English language clinical empirical studies reporting systemic therapy for patients with NS. Systemic therapy included oral, subcutaneous, and intravenous administered medication. There were no restrictions on age of patients. Abstracts, summaries of meetings, and summaries of oral presentations were manually excluded. We excluded studies if: 1) patients were treated for diseases other than NS; 2) systemic treatment lacked specification of type of medication, treatment duration, or treatment related outcome, and 3) if no full text was available.

### 2.3 Study Selection

Duplicates were removed from the identified articles. Two reviewers (AN; SP or TN) independently assessed titles and abstracts for potential eligibility, using Rayyan software (43). Potentially eligible studies were reviewed in full text. All references from included articles were manually screened for additional eligible articles. Any disagreements between reviewers were resolved by discussion with a third reviewer (VD). If studies were considered eligible but lacked specific details, authors were contacted to provide these details. If necessary data could not be provided, studies were excluded.

### 2.4 Data Extraction

Data were extracted independently by two reviewers (AN and TN) using a predefined data extraction template in Excel. Discrepancies were resolved by discussion with a third reviewer (SP, RS, or VD). The following data were extracted if available: author, year of publication, country of inclusion, study design, total number of patients, number of NS patients, number of eligible NS patients, age and sex of patients, given treatment and dosage, duration of treatment, overall treatment effect, primary treatment outcome (effect of treatment on the skin), secondary treatment outcomes (e.g. pruritus, quality of life, frequency of flare-ups), and side effects as provided by the authors. Furthermore, previous and concomitant treatment were noted. Some patients received different systemic treatments consecutively. In this case, each treatment period was described separately and denoted as a treatment case.

### 2.5 Data Analysis and Synthesis

Effect of treatment per received systemic treatment (treatment case) was converted into a 3-level scale reflecting overall treatment effect. Levels were: ‘-’ (worsening of the condition); ‘0’ (no change; or temporary improvement; or a combination of worsening and improvement; or temporary worsening); and ‘+’ (improvement).

For each type of systemic treatment the number of studies, number of treatment cases, number of children, number of females, and number of treatment cases with overall improvement were calculated. Age range and range of treatment duration were collected. Furthermore, reported side effects were summarized per type of treatment.

### 2.6 Quality Assessment

The Joanna Briggs Institute (JBI) critical appraisal checklist for case reports and JBI critical appraisal checklist for case series were used for quality assessment of the case reports and case series respectively (44). Two reviewers (SP and AN) performed the quality assessment. Disagreements were resolved by discussion with a third reviewer (RS). Case series that included only one NS patient were assessed as case reports. Quality assessment was focused on the reported systemic treatments, which was not always the main subject of the included studies. The overall quality of evidence of this review’s primary outcome, effect of treatment on the skin, was assessed using an adapted version of the Grading of Recommendations Assessment, Development and Evaluation (GRADE) methodology (45).

### 2.7 Protocol and Registration

This systematic review was conducted in accordance with the Preferred Reporting Items for Systematic Reviews and Meta-Analyses (PRISMA) statement (46). The review protocol was registered in PROSPERO (ID: 217933) and can be accessed at https://www.crd.york.ac.uk/prospero/display_record.php?RecordID=217933(ref PROSPERO).

## 3 Results

### 3.1 Study Selection

**Figure 1:**
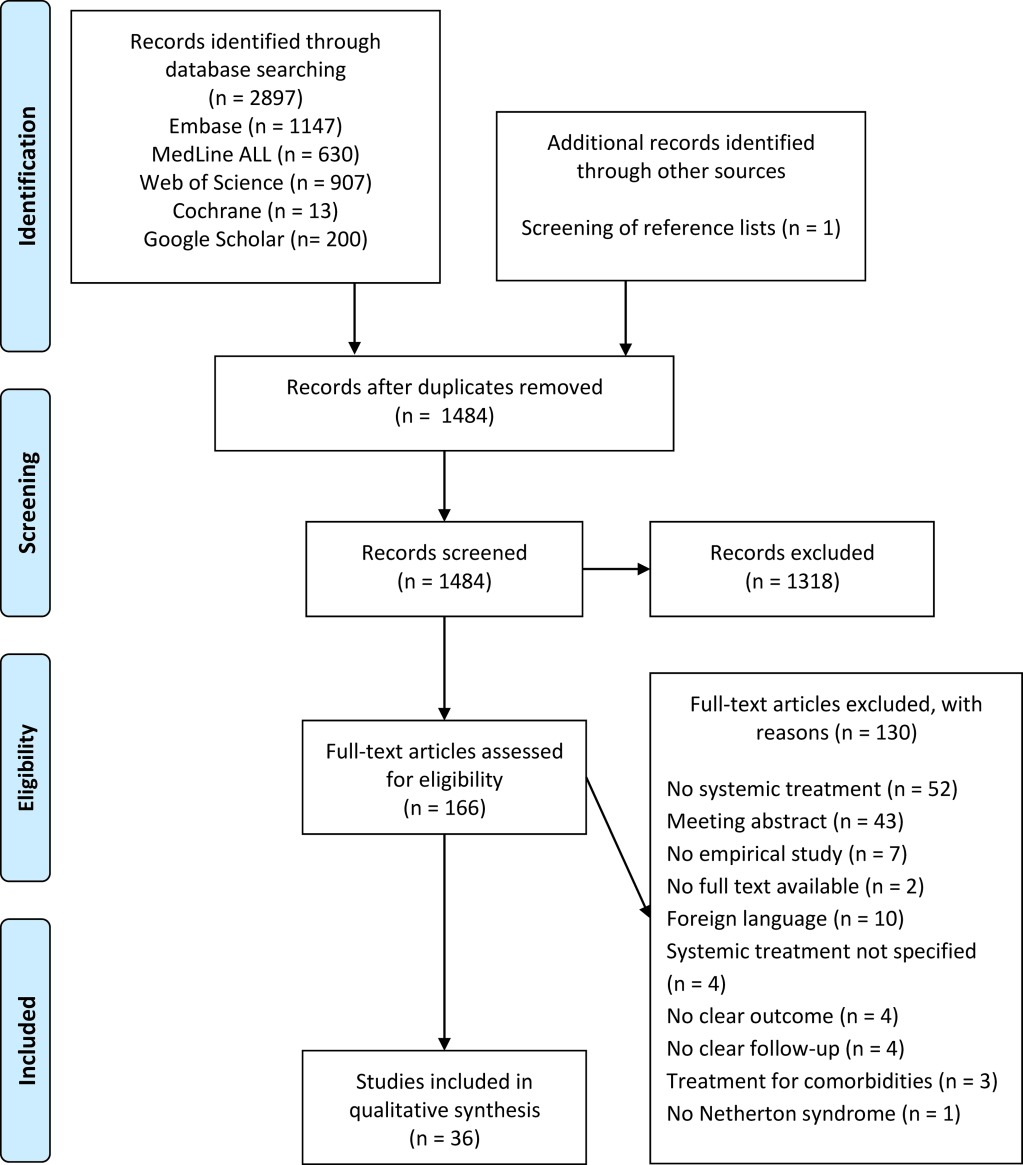
Flowchart of the article selection.

A total of 1484 unique studies were identified through database searching and screening of reference lists, with 166 studies eligible for full-text review based on title and abstract screening. Following the full-text review and approach of authors for missing details, we included 36 studies in the final qualitative analysis (**Figure 1**). Of these articles, 21 studies were case reports and 15 studies were case series. 8 case series only reported one patient with NS. An overview of patient characteristics, study characteristics, and treatment outcomes is provided in **Table 1**. Previous treatments are listed in **Table S1**.

**Table 1:** Characteristics of included studies evaluating systemic therapy in patients with netherton syndrome.

Reference (author, year)	Country^1^	Study design (n^3^)	Eligible NS patients/total NS patients (n)	Age	Sex	Concomitant treatment	Treatment	Dosage	Treatment duration	Overall effect^5^	Primary outcome^6,7,8^	Secondary outcomes^6,7,8^	Reported side effects
Fritsch, 1984 (47)	Austria	Case series^2^ (27)	1/1	23Y	M	NR	Etretinate	1mg/kg/day	4 days	–	Erythroderma: -, burning and oozing erosions face and chest: -	NR	Unclear
Caputo et al., 1984 (37)	Italy	Case series^2^ (2)	1/2	34Y	M	Topical corticosteroids	Etretinate	75mg/day, then discontinued after 4 days. After re-initiation of treatment 75mg/day in the first 2 months, then 50mg/day for 1 month followed by 50mg every other day	4 days, then discontinued. Approximately 2 years after re-initiation of treatment	0	In the first 4 days atopic dermatitis: -. After re-initiation of treatment ichthyosiform lesions after two months: +	Hair growth after two years: +, Ω	No reported side effects
Traupe et al., 1985 (48)	Germany	Case series^2^ (10)	1/1	12Y	M	NR	Etretinate	Initial dose 0.5mg/kg, followed by a maintenance dose of 0.8 mg/kg	At least 22 months	+	ILC after 10 weeks of treatment: +	NR	Unclear
Greene et al., 1985 (38)	USA	Case report (1)	1/1	20Y	M	Penicillin v potassium for 10 days orally	Isotretinoin	Initial dose of 40mg/day (0.5mg/kg), decreased to 40mg every other day after 3 weeks	15 weeks	0	Initial generalized erythema: -, scaling: - and skin fragility: -. Improvement after 3 weeks: 0	NR	Unclear
Hausser et al., 1989 (49)	Germany	Case report (1)	1/1	15Y	F	NR	Acitretin	Initial dose of 35mg/day, decreased to 10 mg every 2nd day after 2 months.	At least 11 months	0	Skin lesions after 4 weeks: +, atopic dermatitis after 8 weeks: -, regression of skin lesions after 11 months: +	After 11 months hair growth: +, hair shaft defects: +, §	Unclear
Groves et al., 1995 (39)	Belgium	Case series^2^ (2)	1/2	11Y	F	PUVA	Etretinate	25 mg/day	8 years	+	Scaling after 6 weeks: +, intensity of erythema: 0	NR	Mild cheilitis
Braun et al., 1997 (50)	Switzerland	Case report (1)	1/1	NR	F	NR	Cyclosporine	Initial dose of 3mg/kg/day, increased to 4 mg/kg/day after 2 months	3 months	0	Skin lesions: 0	NR	Unclear
El Shabrawi et al., 2004 (51)	Austria	Case report (1)	1/1	29Y	M	PUVA	Acitretin	25mg/day	2 weeks	–	Skin condition: -, erythrodermic flare: -	NR	Unclear
Lazaridou et al., 2009 (52)	Greece	Case report (1)	1/1	14Y	M	NR	Isotretinoin	0.2mg/kg	6 months	+	Skin lesions: +	NR	Mild skin dryness, no other reported side effects
Renner et al., 2009 (29)	USA	Case series (9)	3/9^4^	6Y	M	NR	IVIG	0.4g/kg/month	2 years	+	Inflammation: +	Itchting: +, hair thickness: +, scalp condition: +, number of missed school days: +, number of doctor’s visits reduced: +, infection: +, overall quality of life: +, height and weight: +, ¥	Unclear
				2,5Y	M	NR	IVIG	0.4g/kg/month	1 year	+	Inflammation: +	Itchting: +, hair thickness: +, scalp condition: +, number of missed school days: +, number of doctor’s visits reduced: +, infection: +, overall quality of life: +, height and weight: +, ¥	Unclear
				7Y	M	NR	IVIG	0.4g/kg/month	6 months	+	Inflammation: +	Itchting: +, hair thickness: +, scalp condition: +, number of missed school days: +, number of doctor’s visits reduced: +, infection: +, overall quality of life: +, height and weight: +, ¥	Unclear
Fontao et al., 2011 (53)	Switzerland	Case report (1)	1/1	25Y	F	NR	Infliximab	5mg/kg in weeks 0-2-6, then continued every 4 weeks	2 years	+	Clinical improvement after second infusion: +, after 12 infusions inflammatory skin lesions: +, xerosis: 0, ichthyosis: 0	Number of infections: +, §¥	No reported side effects
Gallagher et al., 2012 (54)	USA	Case series^2^ (2)	1/1	at least 4M	F	NR	SCIG	NR	47 weeks	+	Ichthyosis: +	Hair thickness: +, pruritus: +, failure to thrive: +, number of illnesses: +, overall quality of life: +, ¥	Local reaction including mild swelling. One SBI (Escherichia coli urinary tract infection) occurred. No serious adverse events were reported
Maatouk et al., 2012 (35)	Lebanon	Case report (1)	1/1	16Y	F	NR	Isotretinoin	0.2mg/kg/day	At least 1-2 months	–	Skin condition: -	NR	Unclear
Small et al., 2016 (55)	USA	Case series (2)	2/2	16Y	F	NR	IVIG	500 mg/kg monthly	At least 3 months	+	After 3 infusions erythema: +, pustulation: +, scale:+	Pruritus: +, heat tolerance: +, flares: +, secondary infections: +	Unclear
				10Y	M	NR	IVIG	500 mg/kg monthly	At least 3 months	+	After 3 infusions erythema: +, pustulation: +, scale:+	Pruritus: +	Unclear
Yalcin et al., 2016 (56)	Turkey	Case report (1)	1/1	20Y	M	H1 (Chlorphenoxamine, 10 mg)/H2 (Rantidine, 50 mg) blockers (intramuscular) and pulse prednisolone 250 mg once in a week for 4 weeks	Omalizumab	400mg/kg	4 months	+	Clinical improvement: +, histologically reepithelialization below the necrotic epidermis after 2 weeks: +	Use of steroid treatment: +, ¥	Unclear
Roda et al., 2017 (57)	Portugal	Case report (1)	1/1	19Y	F	NR	Infliximab	5mg/kg in weeks 0-2-6, then continued every 8 weeks	(at least) 22 weeks	+	Clinical improvement after the second infusion: +, inflammatory lesions: +, desquamation: +. At week 22 inflammatory lesions: +	Pruritus: +, hair strength and hair length: +	Unclear
Eränkö et al., 2018 (32)	Finland	Case series (11)	3/11	11Y	NR	Topical emollients, topical corticosteroids	IVIG	400 mg/kg/month	11 months	+	Skin condition: +, erythema: +.	Pruritus: +, flares: +, tolerance to topical emollients: +, decrease in use of emollient and topical corticosteroids, hair growth: +, hair thickness: +, skin and other infections: +, GER symptoms: 0, ¥	Unclear
				10Y	NR	Topical emollients, topical corticosteroids	IVIG	400 mg/kg/month	5 months	+	Skin condition: +, erythema: +.	Pruritus: +, flares:+, tolerance to topical emollients: +, decrease in use of emollient and topical corticosteroids, hair growth: +, hair thickness: +, allergic symptoms: +, GER: +, ¥	Unclear
				17Y	NR	NR	IVIG	400 mg/kg/month	6 months	0	Lack of clear benefits: 0	¥	Unclear
Leung et al., 2018 (58)	Malaysia/Canada	Case report (1)	1/1	8Y	M	Topical moisturizer	Acitretin	0.25mg/kg, reduced to 0.12mg/kg after 6 months for another 6 months	12 months	+	In 2 months skin lesions: +, after 6 months erythema: + and scaling: +	After 2 monthts pruritus: +, after 6 months hair growth and hair condition: +	No reported side effects
Onnis et al., 2018 (59)	France	Case series^2^ (13)	1/1	28Y	F	NR	Alitretinoin	Initial dose 10mg/day, increased to 30mg/day after 1 month	6 months	+	Efficacy on prominent signs: +. **VAS** scale before-after treatment: + (6-4); **VAS** erythema: + (8-3), **VAS** palmoplantar keratoderma: + (3-2)	¥	Benign intracranial hypertension, which completely resolved after withdrawal of alitretinoin
Özyurt et al., 2019 (60)	Turkey	Case report (1)	1/1	4Y	M	Topical moisturizers, topical corticosteroids	Acitretin	0.5mg/kg/day	At least 1 year	+	Beneficial effect after 1 year: +, ILC: +	Flare-ups of eczema: +, need to use topical corticosteroids: +	No reported side effects
Yadav et al., 2019 (61)	India	Case report (1)	1/1	23Y	M	Petroleum jelly	Acitretin	25mg/day	2 weeks	–	Within 2 weeks skin condition: -, scaling: -, redness: -	NR	Unclear
Aktas et al., 2020 (62)	Turkey	Case report (1)	1/1	40Y	F	NR	I) IVIG	I) 2 g/kg/month	I) 6 months	I) +	I) Some improvement: +	I) NR	I) Unclear
							II) Dupilumab	II) Initial dose 600mg, followed by 300mg biweekly	II) 3 months	II) 0	II) After 6 weeks eczematous lesions: +, by week 10, lesions: -	II) After 6 weeks pruritus: +, by week 10 pruritus: -	II) Conjunctivitis, treated with topical antibiotics
Andreasen et al., 2020 (63)	Denmark	Case report (1)	1/1	43Y	M	NR	Dupilumab	Initial dose 600mg, followed by 300mg biweekly	6 months	+	After 4 weeks **EASI**: + (22.6-5.3), after 6 months NS minimum: +	After 4 weeks **DLQI**: + (19-2), **POEM**: + (15-9), ¥	Unclear
Blanchard et al., 2020 (64)	USA	Case report (1)	1/1	at least 16Y	M	I) NR	I) IVIG	I) 3 monthly infusions at 0.5g/kg	I) At least 3 months	I) 0	I) Temporary improvement of truncal rash: +	I) NR	I) Unclear
						II) NR	II) Adalimumab	II) 40 mg every other week starting 1 week after an 80mg loading dose	II) At least 6 months	II) 0	II) Intitial response of the facial rash: +, efficacy within 6 months-	II) NR	II) Unclear
						III) Moisturizer and tretinoin 0.025% cream	III) Secukinumab	III) 300mg weekly in the first month, followed by 300mg monthly	III) Almost 3 years	III) +	III) After 4 weeks facial and truncal rash: +. After 3 years facial erythema: +	III) Flare: +	III) Unclear
Barbieux et al., 2020 (65)	France	Case series (3)	3/3	29Y	F	Emollients, topical corticosteroids, calcineurin inhibitors (6M), anti-histamines, oral antibiotics, alitretinoin (5M), thermal cures	Ixekizumab	Starting dose of 2x80mg followed by bi-monthly subcutaneous injections of 80mg during 12 weeks and then monthly injections of 80mg during 12 weeks	6 months	+	In the first 12 weeks: cutaneous improvement: +, oozing: +, scaling: +, **IASI-S**: +. After 6 months **IASI-E**: 0, **IASI-S**: +	In the first 12 weeks: flares: +, pruritus: +, reduction in use of topical corticosteroids: +, **DLQI**: +. At 6 months **DLQI**: +, pruritus: +, §¥	Unclear
				30Y	M	Emollients, topical and oral corticosteroids, anti-histamines, thermal cures	Ixekizumab	Starting dose of 2x80mg followed by bi-monthly subcutaneous injections of 80mg during 12 weeks and then monthly injections of 80mg during 12 weeks	6 months	0	In the first 12 weeks: cutaneous improvement: +, oozing: +, scaling: +, **IASI-S**: +. After 6 months **IASI-E**: 0, **IASI-S**: 0	In the first 12 weeks: flares: +, pruritus: +, reduction in use of topical corticosteroids: +, **DLQI**: +. At 6 months **DLQI**: 0, pruritus: 0, §¥	Unclear
				20Y	F	Emollients, topical corticosteroids, calcineurin inhibitors, anti-histamines, IVIG (8M), oral antibiotics	Ixekizumab	Starting dose of 2x80mg followed by bi-monthly subcutaneous injections of 80mg during 12 weeks and then monthly injections of 80mg during 12 weeks	6 months	+	In the first 12 weeks: cutaneous improvement: +, oozing: +, scaling: +, **IASI-S**: +. After 6 months **IASI-E**: 0, **IASI-S**:+	In the first 12 weeks: flares: +, pruritus: +, reduction in use of topical corticosteroids: +, **DLQI**: +. At 6 months **DLQI**: +, pruritus: +, §¥	Unclear
Dabas et al., 2020 (66)	India	Case series^2^ (6)	1/2	13Y	F	Emollients, antihistamines	I) Acitretin	I) 0.3mg/kg/day	I) 2 months	I) 0	I) Improvement: 0	I) NR	I) Unclear
							II) IVIG	II) Monthly doses of 0.4g/kg/day	II) 6 months	II) +	II) Erythema: + and scaling: +	II) NR	II) 6 months after IVIG treatment: persistent headache caused by thrombosis of left sigmoid and transverse sinus
Luchsinger et al., 2020 (40)	Switzerland	Case series (4)	4/4	21Y	M	Topical corticosteroids	Secukinumab	Weekly from baseline until 4 weeks, then monthly dose (Dosage mentioned as mg/kg based on weight categories, weight and total dosage per patient not reported)	3 months	+	Cutaneous improvement after 2 doses: +. After 3 months **IASI**, **IASI-S**, **IASI-E**: +.	After 3 months **DLQI**: +, **5-D itch scale**: +, use of topical steroids: +, night sleep: +	Onychomycosis due to candida albicans occurred in 2 patients, common viral warts occurred in 1 patients. Both children developed acute pruritic palmoplantar eczematous reaction, refractory to topical corticosteroids
				27Y	F	Topical corticosteroids	Secukinumab	Weekly from baseline until 4 weeks, then monthly dose	12 months	+	Cutaneous improvement after 2 doses: +. After 3 and 6 months **IASI**, **IASI-S**, **IASI-E**: +.	After 3 and 6 months **DLQI**: +, **5-D itch scale**: +, use of topical steroids: +, night sleep: +	
				9Y	M	Topical corticosteroids	Secukinumab	Weekly from baseline until 4 weeks, then monthly dose	8 months	+	Cutaneous improvement after 2 doses: +. After 3 and 6 months **IASI**, **IASI-S**, **IASI-E**: +.	After 3 and 6 months **DLQI**: +, **5-D itch scale**: +, chronic blepharitis: +, recurrent otitis externa: +, use of topical steroids: +, night sleep: +, continued weight gain: +, growth rate: +	
				9Y	M	Topical corticosteroids	Secukinumab	Weekly from baseline until 4 weeks, then monthly dose	7 months	+	Cutaneous improvement after 2 doses: +. After 3 and 6 months **IASI**, **IASI-S**, **IASI-E**: +.	After 3 and 6 months **DLQI**: +, **5-D itch scale**: +, chronic blepharitis: +, skin infections: +, use of topical steroids: +, night sleep: +, use of topical corticosteroids, continued weight gain: +, growth rate: +	
Luchsinger et al., 2020 (67)	Switzerland	Case series^2^ (13)	1/1	NR	M	IVIG, anithistamines	Alitretinoin	0.5 mg/kg/day	8 months	0	Erythema: 0, other skin findings: 0	NR	Unclear
Orlova et al., 2020 (68)	Russia	Case report (1)	1/1	29Y	F	Unna cream (lanolin), Venofer (iron hydroxide complex III with sucrose), antihistamines, Ketotifen 2 mg/day, Chloropyramine, Omeprazole, Amitriptyline 12.5 mg/day	Systemic prednisolone	90mg per day IV days 1-5; 60 mg per day, days 6-11; 30 mg per day, days 12-15	15 days	+	Skin lesions in 1.5 weeks: +	NR	Unclear
Steuer et al., 2020 (69)	USA	Case report (1)	1/1	32Y	F	Topical 0.1% tacrolimus ointment, tazarotene cream, desonide ointment, emollient moisturizer	Dupilumab	Initial dose 600mg, followed by 300mg biweekly	18 months	+	After 10 months: overall disease severity: +, affected **BSA**: + (reduced by 50%). After 18 months: continued improvement: +	Within 2 months: **NRS** itch: + (9-2). After 10 months functioning: +, travel: +	Unclear
Süßmuth et al., 2020 (70)	Germany	Case series (2)	2/2	12Y	F	Growth hormone, Vitamine D	Dupilumab	Initial dose 600mg, followed by 300mg every 4 weeks, then after 4 months therapy was intensified to 300mg every 2 weeks	12 months	+	After 4 months **NASA**: + (33-11.7), **PGA**: + (4-2), erythema: +, scaling: +. After 12 months: **NASA**: + (33-11.7), **PGA**: + (4-2)	**NRS** pruritus 4 months: + (8-3). After 12 months **NRS** pruritus: + (8-2). Overall frequency of cutaneous infections: +, ¥	Bacterial superinfection which led to temprary increase of NASA after 10 months, treated with topical antiseptics
				8Y	M	I) NR	I) SCIG	I) NR	I) 5 years	I) +	I) Clinically more or less beneficial: +	I) Pruritus: 0	I) Unclear
						II) SCIG	II) Dupilumab	II) 300 mg every 4 weeks	II) 10 months	II) +	II) **NASA** after 10 months: + (50.5-18). **PGA** 10 months: + (4-2), erythema: +, scaling: +	II) **NRS** pruritus 10 months: + (8-3)	II) No reported side effects
Volc et al., 2020 (71)	Austria	Case report (1)	1/1	15Y	F	3-monthly injections of medroxyprogesterone acetate	Ustekinumab	45 mg (0.75mg/kg)	At least 1 year	+	After 4 weeks skin symptoms: +, eczematous areas and psoriasiform lesions: +, After 1 year well-doing: +	Signs of Cushing syndrome: +.	Unclear
Zelieskova et al., 2020 (72)	Slovakia	Case report (1)	1/1	12M	M	NR	SCIG	subcutaneous 20% immunoglobulin substitution (SCIg) 1g every 2 weeks (200mg/kg/month)	At least 1 year	+	Generalized erythroderma: +, ichthyosis: +	Weight gain: +, respiratory morbidity: +	No reported side effects
Cicek et al., 2021 (73)	Turkey	Case report (1)	1/1	6M	M	NR	Infliximab	5mg/kg given in weeks 0-2-6, then continued every 4 weeks	1 year	+	After third infusion: skin and scalp: +, after 1 year of treatment, skin and scalp rash: +	NR	No reported side effects
Murase et al., 2021 (41)	Japan	Case series (2)	2/2	32Y	F	NR	Dupilumab	Initial dose of 600mg, followed by 300mg every 2 weeks	6 months	+	After 6 months **EASI**: + (61.8-12.9), **IGA**: + (4-2), **CIS**: + (49.2-21)	1 day after injection: pruritus +, After 6 months: **VAS** itching: + (7-3), hair growth: +, hair strength: +, **HAP**: +, **HSD**: +, ¥	No reported side effects
				17Y	F	NR	Dupilumab	Initial dose of 600mg, followed by 300mg every 2 weeks	6 months	+	After 6 months **EASI**: + (35.6-12.4), **IGA**: + (4-2), **CIS**: + (39-30.5)	1 day after injection: pruritus +, After 6 months **VAS** itching: + (5-3), hair growth: +, hair strength: +, **HAP**: 0, **HSD**: 0, ¥	No reported side effects
Zhang et al., 2021 (74)	China	Case report (1)	1/1	3Y	M	Topical corticosteroids	IVIG	500 mg/kg/month	At least 2 months	+	Eruptions: +	NR	Unclear

NS, Netherton syndrome; N, number; M, male; F, female; Y,Year; M,Month; NR, not reported; USA, United States of America; NASA, Netherton Area Severity Assessment; PGA, Physician Global Assessment; VAS, Visual Analogue Scale; NRS, Numerical Rating Scale; IGA, Investigator Global Assessment; IASI, Ichthyosis Area and Severity Index; IASI-S, Ichthyosis Area and Severity Index - Scaling; IASI-E, Ichthyosis Area and Severity Index - Erythema; EASI, Eczema Area and Severity Index; BSA, Body Surface Area; DLQI, Dermatology Life Quality Index; POEM, Patient Oriented Eczema Measure; CIS, Clinical Ichthyosis Score; HAP, Hair area percentage; HSD, Hair shaft diameter; ILC, Ichthyosis Linearis Circumflexa; IVIG, intravenous immunoglobulins; SCIG, subcutaneous immunoglobulins; SBI, serious bacterial infection; PUVA, psoralen-UVA- therapy; GER, gastro-esophageal reflux; mg, milligrams; g, grams, kg, kilograms; 1= country of patient inclusion; 2 = assessed as case report since only one NS patient was included; 3= total number of patients included in the study; 4 = 5 patients treated with IVIG, but only in 3 patients treatment duration was described and thus 3 patients were eligible for inclusion; 5= ‘+’ indicates improvement of the skin condition, ‘-’ indicates worsening of the skin condition, ‘0’ indicates no change; or temporary improvement; or a combination of worsening and improvement; or temporary worsening of the skin condition; 6= ‘+’indicates improvement, ‘0’ indicates no change, and ‘-’ indicates worsening; 7= if absolute numbers were mentioned in the study these are presented as (before-after); 8= measurement instruments are shown in bold; §= skin biopsy measurements taken prior to and after treatment; ¥= blood measurements taken prior to and after treatment; Ω= urinary amino acids levels measurements taken prior to and after treatment.

### 3.2 Patient Characteristics

Overall, 48 patients with NS treated with systemic agents were included. The study sample consisted of 19 adults and 27 children. The youngest patients were a few months old, the oldest patient was 43 years of age. In two patients, age was not reported (50, 67). 25 patients were male and 20 patients were female. In three patients, sex was not reported (32).

### 3.3 Summary of Systemic Treatment

**Figure 2:**
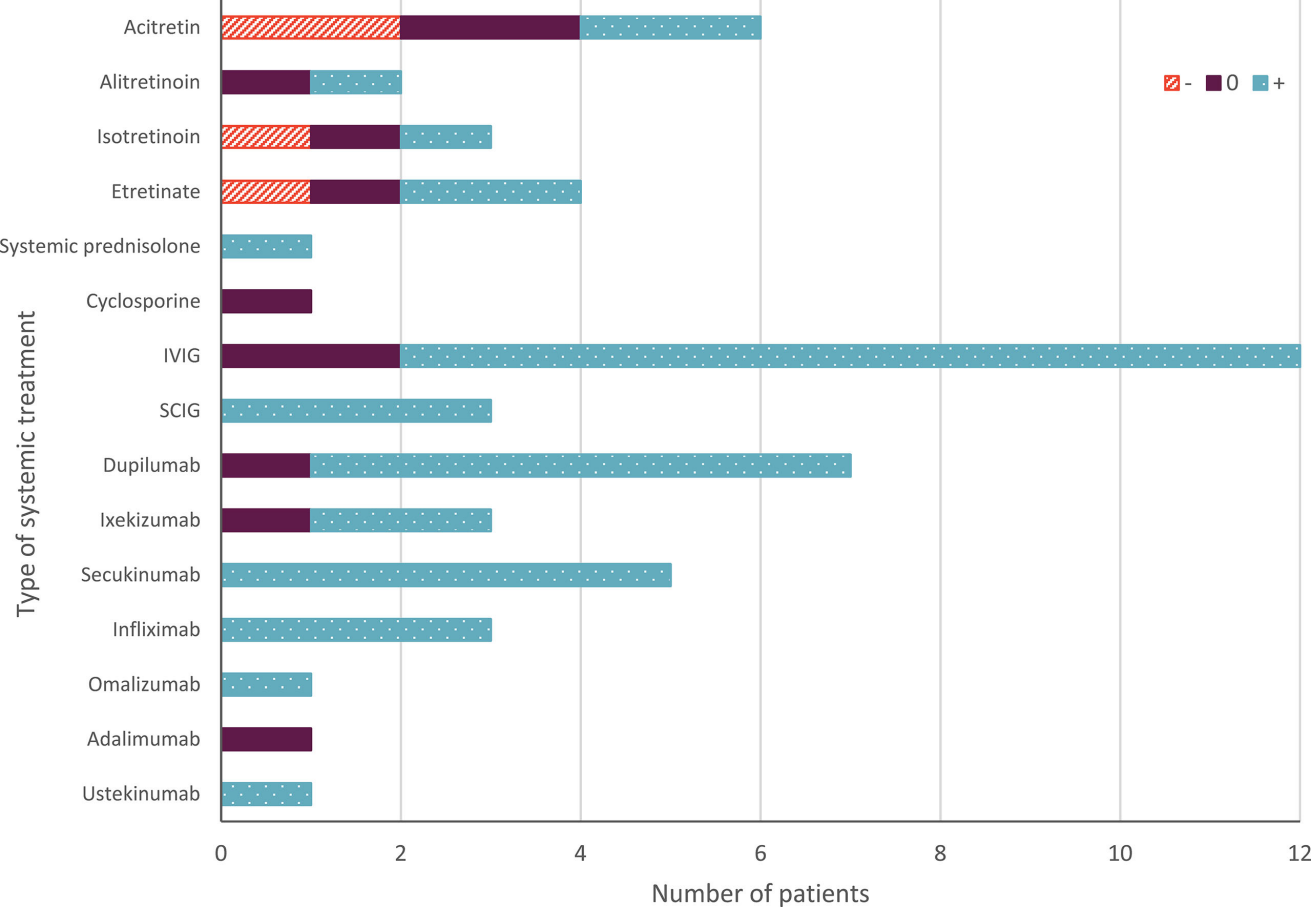
Overall effect of systemic treatment in Netherton syndrome. The orange upward diagonal lines indicate worsening (-) the skin condition; blue with white dots indicates improvement (+) of the skin condition. Purple indicates no change; or temporary improvement; or a combination of worsening and improvement; or temporary worsening (0). The number of patients on the X-axis indicates absolute numbers. IVIG, intravenous immunoglobulins; SCIG, subcutaneous immunoglobulins.

An overview of all reported therapies is presented in **Tables 2**, **S2** and **Figure 2**. Overall, 15 different types of systemic therapies were investigated, belonging to 5 treatment groups. Included treatment groups were systemic retinoids (n= 15), systemic prednisolone (n=1), cyclosporine (n=1), immunoglobulins (n=15), and biologicals (n=21). Of all therapies, intravenous immunoglobulins (IVIG; n=12) and dupilumab (n=7) were most studied.

**Table 2:** Overview of systemic treatments.

Medication	Subgroup medication	Cases (n)^1,2^	F/Total (n)	C/Total (n)	Age range	Treatment duration (range)	Overall effect +/Total^7^	Side effects	Source (references)
Retinoids	Acitretin	6	2/6	4/6	4-28 years	2 weeks - 1 year	2/6	NR	Hausser et al. (49), El Shabrawi et al. (51), Leung et al. (58), Özyurt et al. (60), Yadav et al. (61), Dabas et al. (66)
	Alitretinoin	2	1/2	0/2^4^	28 years^4^	6 months - 8 months	1/2	Benign intracranial hypertension	Onnis et al. (59), Luchsinger et al. (67)
	Isotretinoin	3	1/3	2/3	14-20 years	1-2 months - 6 months	1/3	Mild skin dryness	Lazaridou et al. (52), Maatouk et al. (35), Greene et al. (38)
	Etretinate	4	1/4	2/4	11-34 years	4 days- 8 years	2/4	Mild cheilitis	Fritsch (47), Caputo et al. (37), Groves et al. (39), Traupe et al. (48)
Other	Systemic prednisolone	1	1/1	0/1	29^5^	15 days^5^	1/1	NR	Orlova et al. (68)
	Cyclosporine	1	1/1	NR	NR	3 months^5^	0/1	NR	Braun et al. (50)
Immunoglobulins	IVIG	12(7)	3/9^3^	11/12	2,5-40 years	2 months - 2 years	10/12	Persistent headache caused by thrombosis of left sigmoid and transverse sinus 6 months after IVIG in 1 patient	Renner et al. (29), Small et al. (55), Eränkö et al. (32), Dabas et al. (66), Zhang et al. (74), Aktas et al. (62), Blanchard et al. (64)
Immunoglobulins	SCIG	3	1/3	3/3	4 months - 8 years	47 weeks - 5 years	3/3	Local reaction including mild swelling; SBI (Escherichia coli urinary tract infection)	Gallagher et al. (54), Süßmuth et al. (70), Zelieskova et al. (72)
Biologicals	Dupilumab	7(5)	5/7	3/7	8-43 years	3 months - 18 months	6/7	Conjunctivitis; bacterial superinfection	Andreasen et al. (63), Steuer et al. (69), Süßmuth et al. (70), Murase et al. (41), Aktas et al. (62)
	Ixekizumab	3(1)	2/3	0/3	20-30 years	6 months^6^	2/3	NR	Barbieux et al. (65)
	Secukinumab	5(2)	1/5	3/5	9-27 years	3 months - 3 years	5/5	Onychomycosis due to *candida albicans*, common viral warts, acute pruritic palmoplantar eczematous reaction	Blanchard et al. (64), Luchsinger et al. (40)
	Infliximab	3	2/3	1/3	6 months - 25 years	22 weeks - 2 years	3/3	NR	Roda et al. (57), Cicek et al. (73), Fontao et al. (53)
	Omalizumab	1	0/1	0/1	20 years^5^	4 months^5^	1/1	NR	Yalcin et al. (56)
	Adalimumab	1	0/1	1/1	16 years^5^	6 months^5^	0/1	NR	Blanchard et al. (64)
	Ustekinumab	1	1/1	1/1	15 years^5^	1 year^5^	1/1	NR	Volc et al. (71)

1= The total of cases is not equal to the total number of included patients. One patient can be counted as multiple treatment cases if the patient has received multiple types of systemic treatment consecutively; 2= if the number of cases differs from the number of studies this is shown as: number of cases (number of studies); 3= of three patients sex was not reported; 4= age of 1 of the patients is not reported; 5= data based on one reported patient; 6= treatment duration was 6 months for all three patients; 7= this column shows the number of patients with improvement during treatment/total number of patients; 8= studies either stated that no side effects occurred or the occurrence of side effects remains unclear: see **Table 1**. IVIG, Intravenous immunoglobulins; SCIG, Subcutaneous immunoglobulins; n, Absolute number; F, Female; C, Child; NR, Not reported.

In 4 patients the consecutive use of two or more systemic therapies was reported, for which outcome and treatment duration were described per therapy and therefore count as multiple cases (62, 64, 66, 70). In total 53 treatment cases were reported in 48 patients. Many patients received previous topical and systemic therapy, but this was not consistently reported in all studies (see **Table S1**). In 18 patients simultaneous use of topical treatments including corticosteroids was reported during treatment with the systemic agents (see **Table 1**). 7 patients received multiple systemic treatments for NS simultaneously which sometimes prevented interpretation of treatment results (38, 56, 65, 67, 70).

Overall, treatment duration varied widely. In most treatment cases outcomes were only assessed short-term, with treatment durations of 6 months or shorter. Only 30% of the cases had treatment durations of one year or longer. Duration of treatment in patients with retinoids varied from 4 days to 2 years, with one outlier having a treatment duration of at least 8 years. In patients treated with immunoglobulins and biologicals the treatment duration ranged between 2 months to 5 years and 3 months to 3 years, respectively (see **Tables 1**, **2** and **S2**).

### 3.4 Outcomes

Outcomes were measured in numerous ways using both descriptive methods and standardised measurement instruments (see **Tables 1**, **2**).

#### 3.4.1 Primary Outcome: Skin

All studies reported the effect of treatment on the skin. Most studies used descriptive methods, and focused on different aspects of the skin (e.g. erythema, scaling), resulting in a large inter-study variability. A total of 10 different measurement instruments were reported in 7 studies, including the Eczema Area and Severity Index (EASI), Ichthyosis Area and Severity Index (IASI), Ichthyosis Area and Severity Index – Scaling (IASI-S), Ichthyosis Area and Severity Index – Erythema (IASI-E), Netherton Area Severity Assessment (NASA), Physician Global Assessment (PGA), Clinical Ichthyosis Score (CIS), Investigator Global Assessment (IGA), Visual Analogue Scale (VAS; e.g. for erythema), and Body Surface Area (BSA) (see **Table 1**).

#### 3.4.2 Secondary Outcomes

22 studies reported the effect of systemic treatment on secondary outcomes (see **Table 1**). Pruritus or itch, effects on hair, use of topical corticosteroids, infections, and quality of life were mostly reported. Other notable outcomes were height and weight (especially in children), and frequency of flare-ups. Most studies used descriptive methods. A total of 7 different measurement instruments were reported for the secondary outcomes: 5-D Itch Scale, Patient Oriented Eczema Measure (POEM), Dermatology Life Quality Index (DLQI), Numerical Rating Scale (NRS) and the Visual Analogue Scale (VAS) for pruritus, Hair Area Percentage (HAP), and Hair Shaft Diameters (HSD) (see **Table 1**).

### 3.5 Overall Effect

Most reported treatments showed improvement of the patient’s condition (38/53 treatment cases). Immunoglobulins showed improvement in 13/15 cases, and biologicals in 18/21 of the cases. In cases treated with retinoids, both worsening (4/15 cases) and improvement (6/15 cases) of the skin condition was reported (see **Tables 2**, **S2** and **Figure 2**).

### 3.6 Safety of Systemic Treatment

Occurrence of adverse events or side effects was reported in 8 of the 36 studies (see **Tables 1** , **2**). Overall, 11 adverse events or side effects were reported, such as cheilitis, local swelling, conjunctivitis, and onychomycosis. The highest number of side effects (3 types of events) was reported in patients treated with secukinumab, who experienced onychomycosis due to *Candida albicans*, common viral warts, and palmoplantar eczematous reactions (40) (see **Table 2**). In 9 patients (8 studies) it was reported that no side effects had occurred (see **Table 1**).

### 3.7 Study Quality Assessment

The results of the JBI critical appraisal checklist for case reports and case series are summarized in **Tables 3**, **4** respectively. Overall, study quality of the included studies was moderate-to-good. Most of the case reports scored 6 out of 8 quality criteria or higher (see **Table 3**). All case series scored 6 out of 10 quality criteria or higher (see **Table 4**). The quality of evidence of the effect of systemic treatment on the skin according to the GRADE methodology is reported in **Table 5**.

**Table 3:** Quality assessment of the included case reports using the Joanna Briggs Institute (JBI) Critical Appraisal Checklist for Case Reports.

Reference (author, year)	A1	A2	A3	A4	A5	A6	A7	A8	Total yes (max. 8)
Fritsch, 1984 (47)	Yes	No	No	No	yes	Yes	Unclear	Yes	4
Caputo et al., 1984 (37)	Yes	No	Yes	Yes	Yes	Yes	Yes	No	6
Traupe et al., 1985 (48)	Yes	No	No	Yes	Yes	Yes	Unclear	Yes	5
Greene et al., 1985 (38)	Yes	Yes	Yes	Yes	Yes	Yes	Unclear	Yes	7
Hausser et al., 1989 (49)	Yes	No	Yes	Yes	Yes	Yes	Unclear	Yes	6
Groves et al., 1995 (39)	Yes	No	Yes	Yes	Yes	Yes	Yes	Yes	7
Braun et al., 1997 (50)	No	No	No	No	Yes	Yes	Unclear	No	2
El Shabrawi et al., 2004 (51)	No	No	Yes	Yes	Yes	Yes	Unclear	Yes	5
Lazaridou et al., 2009 (52)	Yes	No	Yes	Yes	Yes	Yes	Yes	Yes	7
Fontao et al., 2011 (53)	Yes	Yes	Yes	Yes	Yes	Yes	Yes	Yes	8
Gallagher et al., 2012 (54)	Yes	No	No	Yes	Yes	Yes	Yes	Yes	6
Maatouk et al., 2012 (35)	Yes	Yes	No	Yes	Yes	Yes	Unclear	Yes	6
Yalcin et al., 2016 (56)	Yes	No	Yes	Yes	Yes	Yes	Unclear	Yes	6
Roda et al., 2017 (57)	Yes	Yes	Yes	Yes	Yes	Yes	Unclear	Yes	7
Leung et al., 2018 (58)	Yes	Yes	Yes	Yes	Yes	Yes	Yes	Yes	8
Onnis et al., 2018 (59)	Yes	No	No	No	Yes	Yes	Yes	Yes	5
Özyurt et al., 2019 (60)	Yes	Yes	Yes	Yes	Yes	Yes	Yes	Yes	8
Yadav et al., 2019 (61)	Yes	No	Yes	Yes	Yes	Yes	Unclear	Yes	6
Aktas et al., 2020 (62)	Yes	No	Yes	Yes	Yes	Yes	Yes	Yes	7
Andreasen et al., 2020 (63)	Yes	Yes	Yes	Yes	Yes	Yes	Unclear	Yes	7
Blanchard et al., 2020 (64)	Yes	Yes	Yes	Yes	Yes	Yes	Unclear	Yes	7
Dabas et al., 2020 (66)	Yes	Yes	Yes	Yes	Yes	Yes	Yes	Yes	8
Luchsinger et al., 2020 (67)	No	No	Yes	Yes	Yes	Yes	Unclear	Yes	5
Orlova et al., 2020 (68)	Yes	Yes	Yes	Yes	Yes	Yes	Unclear	Yes	7
Steuer et al., 2020 (69)	Yes	Yes	Yes	Yes	Yes	Yes	Unclear	Yes	7
Volc et al., 2020 (71)	Yes	Yes	Yes	Yes	Yes	Yes	Unclear	Yes	7
Zelieskova et al., 2020 (72)	Yes	Yes	No	Yes	Yes	Yes	Yes	Yes	8
Cicek et al., 2021 (73)	Yes	Yes	Yes	Yes	Yes	Yes	Yes	Yes	8
Zhang et al., 2021 (74)	Yes	Yes	Yes	Yes	Yes	Yes	Unclear	Yes	7

Answer options include: ‘Yes’, ‘No’, ‘Unclear’ or ‘Not applicable’; A1: Were patient’s demographic characteristics clearly described?; A2: Was the patient’s history clearly described and presented as a timeline?; A3: Was the current clinical condition of the patient on presentation clearly described?; A4: Were diagnostic tests or assessment methods and the results clearly described?; A5: Was the intervention(s) or treatment procedure(s) clearly described?; A6: Was the post-intervention clinical condition clearly described?; A7: Were adverse events (harms) or unanticipated events identified and described?; A8: Does the case report provide takeaway lessons?

**Table 4:** Quality assessment of the included case series using the Joanna Briggs Institute (JBI) Critical Appraisal Checklist for Case Series.

Reference (author, year)	B1	B2	B3	B4	B5	B6	B7	B8	B9	B10	Total yes (max. 10)
Small et al., 2016 (55)	Yes	Yes	Yes	Unclear	Unclear	Yes	Yes	Yes	yes	Not applicable	7
Eränkö et al., 2018 (32)	Yes	Yes	Yes	Yes	Unclear	No	Yes	Yes	No	Yes	7
Barbieux et al., 2020 (65)	Yes	Yes	Yes	Unclear	Unclear	Yes	Yes	Yes	Yes	Yes	8
Luchsinger et al., 2020 (40)	Yes	Yes	Yes	Yes	Unclear	Yes	Yes	Yes	Yes	Yes	9
Süßmuth et al., 2020 (70)	Yes	Yes	Unclear	Unclear	Unclear	Yes	No	Yes	Yes	Yes	6
Renner et al., 2009 (29)	Yes	Yes	Yes	Unclear	Unclear	Yes	Yes	Yes	Yes	Yes	8
Murase et al., 2021 (41)	Yes	Yes	Yes	Unclear	Unclear	Yes	Yes	Yes	Yes	Yes	8

Answer options include: ‘Yes’, ‘No’, ‘Unclear’ or ‘Not applicable’; B1: Were there clear criteria for inclusion in the case series?; B2: Was the condition measured in a standard, reliable way for all participants included in the case series?; B3: Were valid methods used for identification of the condition for all participants included in the case series?; B4: Did the case series have consecutive inclusion of participants?; B5: Did the case series have complete inclusion of participants?; B6: Was there clear reporting of the demographics of the participants in the study?; B7: Was there clear reporting of clinical information of the participants?; B8: Were the outcomes or follow up results of cases clearly reported?; B9: Was there clear reporting of the presenting site(s)/clinic(s) demographic information?; B10: Was statistical analysis appropriate?

**Table 5:** The certainty of evidence for the primary outcome, effect of treatment on the skin, rated using the Grading of Recommendations, Assessment, Development, and Evaluations (GRADE) approach by Murad et al. (45).

Grade domain	Judgement	Concerns about certainty domains
Methodological limitations of the studies	All studies included in this systematic review were case reports or (small) case series. Most studies were of moderate-good quality as assessed by the Joanna Briggs Institute Critical Appraisal Checklist for Case Reports or Series respectively (see **Table 3** and **Table 4**). However, these studies did not report risk of bias items (e.g. blinding of patients or physicians, complete inclusion of participants). Based on study design, we cannot rule out the presence of bias across studies, which is a concern.	Serious
Indirectness	A majority of studies reported the methods that were used to diagnose patients with NS. All studies reported the type of used medication. However, the dosage per kilograms was not always described, which makes comparison between studies difficult. Furthermore, in some studies multiple therapies were used simultaneously. Treatment duration varied highly among studies. Lastly, outcomes were not similarly defined between studies. The treatment outcomes were assessed using different methods and instruments across studies.	Serious
Imprecision	All included studies were case series with a small sample size or case reports. The total sample size was small (n= 48), resulting in even smaller number of patients in treatment subgroups. This can lead to imprecision. However, it remains important to note that NS is a rare disease.	Serious
Inconsistency	The direction and magnitude of effect varied across the different studies reporting treatment with retinoids (see **Table 2**). Patients with improvement, no effect, and worsening of the skin were described. Therefore, we assumed the evidence on retinoids to have serious inconsistency. In most cases that received other therapies, including immunoglobulins and biologicals, improvement of the skin was described. No overall worsening was described, and in few patients no change or temporary change was reported. Therefore we conclude that in these groups, there is some inconsistency in the magnitude of effect, resulting in borderline serious inconsistency.	Serious for retinoids; Borderline serious for immunoglobulins and biologicals
Publication bias	We do not strongly suspect publication bias because both negative and positive trials were published, and the search for studies was comprehensive. Studies that reported negative effects on the skin due to retinoids were mostly older studies. Recent studies mostly reported positive effects on the skin. This is likely due to the fact that most recent studies investigated the effect of immunoglobulins and biologicals, which are newer type of medications. However, we have no certainty that all generated data were fully reported.	May be suspected

Conclusion: very low certainty ⊕OOO.

## 4 Discussion

In this systematic review, we analyzed the use and outcomes of systemic treatment in patients with NS. Despite uncertainties, immunoglobulins and biologicals seem most effective in treatment of NS on both skin-related and secondary outcomes. Other treatments such as retinoids were described to be less effective. NS is a rare disease and although available literature is scarce, we included 36 studies describing 48 patients among which 27 were children. We identified 15 different types of systemic treatment for patients with NS. The primary outcome, effect of treatment on the skin, was reported very heterogeneously using descriptive methods and various measurement instruments, focusing on multiple aspects of the skin. This hinders accurate comparison between studies. Secondary outcomes were not mentioned in all studies, and if mentioned, mainly focused on skin related problems, including pruritus, use of topical corticosteroids, infections, and hair. Quality of life was only reported in 5 studies (29, 40, 54, 63, 65). The strong focus on skin related problems combined with sparse attention for patients’ functioning as a person does not reflect the full range of impact that NS has on patient’s lives (75). Furthermore, side effects were infrequently reported, which may flatter treatment outcomes. Little is known about long-term outcomes of systemic therapy in NS.

### 4.1 Systemic Treatment in NS

#### 4.1.1 Retinoids

Four systemic retinoids (acitretin, alitretinoin, isotretinoin, and etretinate) have been used in NS, with varying results. Retinoids are synthetic analogues of vitamin A that act *via* an anti-keratinizing effect and have been used in the treatment of ichthyosis for decades (76, 77). Despite the fact that retinoids have been used in dermatology for a significant amount of time, the majority of studies had only several months of retinoid use. One single study reported the use of etretinate for at least 8 years, with positive effects on scaling (39). In general, long-term effects and potential side effects of retinoids in NS remain unclear.

In most children and adults treated with retinoids, only skin related outcomes were reported. If secondary outcomes were mentioned, they usually focused on aspects of hair. Most studies reporting outcomes of retinoids in NS were published before *SPINK5* mutation analysis was available or commonly used (78). Therefore, NS diagnosis in these studies was usually based on investigation of the hair to characterize trichorrhexis invaginata (bamboo hair). This may explain the elaborate description of outcomes based on hair in these studies.

There are currently no factors that can predict treatment outcome of retinoids in patients with NS. Although four patients with NS worsened during retinoid therapy, six patients improved, with less scaling, erythema, and pruritus, accompanied by a reduction in use of topical corticosteroids. In one patient treated with isotretinoin, worsening of the skin condition stopped when the dosage was reduced (38). This could be due to the course of the disease, or may indicate that patients with NS benefit from a lower dose. In contrast, several other NS patients improved during therapy with high dose retinoids. However, the effect of treatment dosages on outcome is difficult to determine since weight has been infrequently reported and dosages were reported as either total milligrams per day or milligrams per kilogram.

Considering the anti-keratinizing effect of retinoids, which results in removing scales and thinning of hyperkeratosis, it could be hypothesized that retinoids are effective in NS patients in which scaling is more pronounced compared to erythroderma. However, scaling was not assessed consistently, nor has it been measured using standardized instruments across studies. Therefore, no definitive conclusion can be drawn on the effectiveness of retinoids on certain subgroups of the NS population. Based on the current data and compared to immunoglobulins and biologicals, retinoids could be a secondary choice for therapy.

#### 4.1.2 Systemic Prednisolone

Prednisolone is a synthetic corticosteroid closely related to prednisone (79). Corticosteroids are used in NS for their anti-inflammatory and immunosuppressive properties (79, 80). Short-term systemic prednisolone for 15 days resulted in an improvement of skin lesions in one adult patient (68). Additionally, one adult patient used prednisolone for 4 weeks as concomitant therapy during the 4-month treatment with omalizumab (56). Although clinical improvement was observed in this patient, it is unclear whether the improvement can be attributed to prednisolone alone. Furthermore, six studies reported previous use of systemic corticosteroids in both children and adults with NS (41, 63–66, 71). Based on the mechanism of action and known side effects, systemic corticosteroids might not be the first choice of treatment in NS, but could be useful in flare-ups.

#### 4.1.3 Cyclosporine

Cyclosporine is a calcineurin inhibitor, which can be used as an immunosuppressant in auto-immune and cutaneous diseases, including atopic dermatitis (81). Cyclosporine showed no effect on skin lesions in a single patient of unknown age treated for 3 months (50). Three other patients had been previously treated with cyclosporine, but the effects of treatment were not described (62, 64, 69). Based on one reported patient, we cannot determine whether treatment with cyclosporine is beneficial in patients with NS.

#### 4.1.4 Immunoglobulins

The use of both IVIG and SCIG has been reported in NS and thirteen out of fifteen patients showed clinical improvement after treatment initiation (see **Table 2**).

Immunoglobulins have been used in the treatment of primary immunodeficiencies and chronic inflammatory diseases (82–84). Since NS has been described as a primary immunodeficiency disorder, it has been hypothesized that the disorder may respond to treatment with immunoglobulins (29, 32, 33). Interestingly, most patients showed normal serum IgG levels, suggesting that the potential beneficial effects of immunoglobulin replacement therapy (IGRT) in these patients is merely based on the immunomodulatory mode of actions of IGRT (29, 32, 54, 85). This hypothesis is supported by the increase in NK cell cytotoxicity, changes in proportion of CD cells (including CD4, CD8 and CD16), and normalization of certain lymphocyte subclasses (including memory T cells, transitional and activated B cells and plasmablasts) observed during IVIG therapy (29, 32).

However, the exact mechanism of action remains to be elucidated.

The beneficial effects described in patients on supplemental dosage of IGRT is remarkable, since for immunomodulatory purposes in other inflammatory diseases mostly higher doses are prescribed (83, 85). The only adult NS patient that was described, received IVIG at a dose of 2 grams/kilogram/month, with some clinical improvement (62). The pediatric NS patients receiving IVIG were mostly treated at a dosage of 400-500mg/kg/month which is at supplemental dose (86).

Most NS patients treated with immunoglobulins were children (n=14), and in a majority (n=12) a positive clinical effect was observed during a 2 months to 5 years treatment period. Primary effects were a decrease in inflammation, erythema, scaling, and pustulation of the skin. Furthermore, a decrease in itch and infections, decrease in use of topical steroids, improvement of the quality of hair, and improvement in overall quality of life and functioning was reported (see **Tables 1**, **S2**). Remarkably, in 5 children, aged between 4 months and 2 years, a significant increase in weight and height was noted (29, 54, 72). This effect might be attributed to a general improvement in NS symptoms, thereby enabling growth. Another explanation may be that this is a treatment-specific effect.

Clinical data of immunoglobulin treatment in adult NS patients are lacking. However, based on the mechanism of action, immunoglobulins could be equally effective in children and in adults. The efficacy of immunoglobulins has only been reported in one adult (62). During a 6-month treatment period this adult patient experienced some improvement of her skin condition. To assess the role of immunoglobulins in adult patients, more data is needed.

#### 4.1.5 Biologicals

In all use of biologicals initial improvement of the skin condition in NS was observed, suggesting an important role for targeted therapy in the future. As a consequence of the *SPINK5* gene mutation and LEKTI deficiency, allergic and inflammatory pathways are upregulated in patients with NS (18, 20, 22, 29, 87). Increased activity of both Th2 and Th17 pathway, and high levels of TNF-a and IgE have been observed (18, 20, 22–24, 29, 87–89). In previous studies the effect of seven biologicals has been investigated (see **Table 2**). These biologicals specifically target TNF-a (adalimumab, infliximab), IL-17 (secukinumab, ixekizumab), IL-12 and IL-23 (ustekinumab), IL-4 and IL-13 (dupilumab), and IgE (omalizumab). Dupilumab (n=7) and secukinumab (n=5) were most studied in children and adults with NS (see **Table 2**). In 18 cases treated with biologicals, of which 8 were children, improvement was observed, resulting in less inflammatory lesions, desquamation, and oozing. Also, pruritus, quality of life, hair quality, and sleep improved, and use of topical corticosteroids and occurrence of infections decreased. Similar to the observations in pediatric patients treated with immunoglobulins, one study reported an increased growth rate in two children treated with secukinumab (40). Although these patients were slightly older (both 9 years) than the patients treated with immunoglobulins, this may indicate that growth and weight improve if NS symptoms improve, regardless of the type of medication.

After initial improvement the effectiveness decreased in one out of seven patients receiving dupilumab, one out of three patients receiving ixekizumab and the single one patient receiving adalimumab (62, 64, 65). Barbieux et al. reported that the patient who showed decreased effectiveness of ixekizumab initially presented with erythroderma compared to the other two patients in the study who presented with ichthyosis linearis circumflexa (65). This may indicate that clinical phenotype could be an important factor when considering targeted therapy with biologicals. Furthermore, the varying clinical symptoms that are observed in NS might be caused by differences in pathway activity between patients. This could implicate that individual NS patients might benefit from different treatments, depending on phenotype.

Although current research is shifting towards therapies involving the Th17 pathway, this review shows that both biologicals targeting Th17 and Th2 pathway seem effective in NS. However, reports on the effects of biologicals in NS remain scarce and more importantly dispersed over seven types of biologicals. Furthermore, treatment duration varied between 3 months and 3 years, leaving long-term effects not yet well-established (see **Table 2**). More research is needed to assess the role of different types of biologicals in the treatment of NS. Nonetheless, biologicals seem promising agents for the future treatment of NS.

### 4.2 Outcomes

Large heterogeneity was observed in both primary and secondary outcomes. This hinders interpretation of outcomes and comparison of results between studies. In this review we observed that earlier studies used predominantly descriptive methods to evaluate treatment outcomes, whereas more recent publications employed more standardized measurement instruments, albeit with great heterogeneity within and across therapeutic groups. Therefore, it is difficult to determine the best treatment options for NS patients. Heterogeneity in reporting is probably due to NS being a rare disorder having small clusters or single cases scattered across the globe. International collaboration including consensus on a core outcome set and measurement instruments for NS will overcome this issue and improve the quality of NS research.

### 4.3 Strengths and Limitations

This systematic review included data on systemic treatment in both children and adults, on the short- and long term, focusing not only on the skin, but also on a variety of outcomes related to NS. With this broad focus we strived for equipoise, given all possible systemic treatments for NS. Furthermore, although NS is a rare disease, 53 treatment cases in 48 patients were included.

Although this review used the best available data, there are limitations that should be considered. Most studies were of moderate-good quality as assessed by the JBI critical appraisal checklist for case reports or case series respectively (see **Tables 3** and **4**). However, since all included studies were either case reports or case series with low numbers of patients, the quality of evidence of the effect of treatment on the skin was judged as having very low certainty (see **Table 5**). In a case (series) it is difficult to distinguish between the true treatment effect and the natural disease course of NS. As previously mentioned, large heterogeneity was observed in reported outcomes. This prevents thorough comparison and interpretation of outcomes between studies. Most outcomes were skin-related, which may not reflect the full range of patients’ symptoms or needs. In addition, side effects and adverse events of treatment were often not systematically reported. We cannot rule out that publication bias influenced results, leaving room for error in determining the true response to systemic agents. This publication bias was presumably enlarged as a consequence of our inclusion criterion to include only English language articles.

### 4.4 Future Research

To date, the field of research in NS is evolving. Recently, a randomized controlled trial (RCT) investigating the efficacy and safety of secukinumab in ichthyoses completed its recruitment phase, including five patients with NS (90). Also, a pilot study investigating the efficacy and safety of dupilumab versus placebo in patients with NS is currently ongoing (91). It remains questionable whether classical RCTs, are a suitable, efficient, and ethical approach for evaluating treatment of rare (skin) diseases such as NS. Therefore, other methodologic strategies should become more accepted for these kind of diseases. Interesting strategies are study designs in which cases serve as their own control and repeated measurements are performed. Furthermore, adaptive designs that allow for statistical modification of elements of the RCT design can be used (92).

Another step to improve research in NS, is the adoption of standardized outcomes and instruments. The development of a core outcome set for NS by health care professionals and patients, together with a consensus on measurement instruments for future research, will improve data quality and comparability. Improvement of data quality will spur the development of new systemic treatments for NS.

Future research into pathogenesis and NS phenotypes may help predict effectiveness of treatment and identify targeted treatment for patients with NS. International embedded research is needed to evaluate the effect of targeted (systemic) therapy, including long-term use of immunoglobulins and biologicals. This progress in the field of rare diseases can only be obtained by large scale international collaboration between clinicians, researchers, patients, and industry. Ultimately, a treatment guideline is necessary to facilitate high-quality care for patients with NS throughout life.

### 4.5 Conclusion

Netherton syndrome (NS) is a rare disease, which is reflected in the scarce literature on systemic treatment outcomes in children and adults with NS. 36 case series and reports describing 27 children and 19 adults with NS were identified. Despite low quality of evidence and large heterogeneity in reported outcomes, a general beneficial effect of systemic treatment was found. Immunoglobulins and biologicals showed the most promising results, on skin-related symptoms, pruritus, hair quality, use of topical corticosteroids, infections, and quality of life. Both treatments should be further explored. Future research should first focus on determining a core outcome set and standardized measurement instruments for NS to improve quality of NS research. International cooperation between clinicians, researchers, patients, and industry is needed to develop better care for these patients with high unmet medical needs.

## Data Availability Statement

The original contributions presented in the study are included in the article/**Supplementary Material**. Further inquiries can be directed to the corresponding author.

## Author Contributions

SP, TN, VD, RS, AR, and AN designed the study. SP, AN, and TN were responsible for the formal screening of search results against eligibility criteria, data extraction and risk of bias assessment. Any disagreement was resolved by a discussion with VD or RS as third reviewer. AN, TN, and RS wrote the manuscript. RS supervised the study. All authors (AN, RS, TN, AR, AB, CB, VD, and SP) critically commented on the manuscript. All authors contributed to the article and approved the submitted version.

## Conflict of Interest

The authors declare that the research was conducted in the absence of any commercial or financial relationships that could be construed as a potential conflict of interest.

## Publisher’s Note

All claims expressed in this article are solely those of the authors and do not necessarily represent those of their affiliated organizations, or those of the publisher, the editors and the reviewers. Any product that may be evaluated in this article, or claim that may be made by its manufacturer, is not guaranteed or endorsed by the publisher.
