# Supplementary material for: Outcomes of Systemic Treatment in Children and Adults With Netherton Syndrome: A Systematic Review
Source: Front Immunol. 2022 Mar 30;13:864449. doi: 10.3389/fimmu.2022.864449 (PMC9022473; doi:10.3389/fimmu.2022.864449)
Supplement: Supplementary file 1 [file DataSheet_1.pdf]

Supplementary Material

1 Search strategy

Search: up to 21 July, 2021.

Embase.com

('Netherton disease'/de OR 'serine peptidase inhibitor Kazal type 5'/de OR (netherton* OR SPINK5 OR Kazal-type-5 OR LEKTI OR (Lympho* NEAR/3 Kazal-type NEAR/3 inhibitor*)):ab,ti,kw) NOT ((animal/exp OR animal*:de OR nonhuman/de) NOT ('human'/exp))

Medline (Ovid)

(Netherton Syndrome/ OR Serine Peptidase Inhibitor Kazal-Type 5/ OR (netherton* OR SPINK5 OR Kazal-type-5 OR LEKTI OR Kazal-type-related-inhibitor*).ab,ti,kf.) NOT (exp animals/ NOT humans/)

Web of Science

TS=((netherton* OR SPINK5 OR Kazal-type-5 OR LEKTI OR (Lympho* NEAR/2 Kazal-type NEAR/2 inhibitor*)))

Cochrane Central

(netherton* OR SPINK5 OR Kazal-type-5 OR LEKTI OR (Lympho* NEAR/3 Kazal-type NEAR/3 inhibitor*)):ab,ti,kw

Google Scholar

netherton|SPINK5|“Kazal type 5”|LEKTI|“Lympho|lymphoepithelial Kazal type inhibitor”

netherton|SPINK5|'Kazal type 5'|LEKTI|'Lympho|lymphoepithelial Kazal type inhibitor'

frontiers

Table S1. Previous treatments of included Netherton syndrome patients

Reference (author, year)	Country[1]	Study design	Patients (N)	NS patients (N)	Eligible NS patients (N)	Age	Sex	Previous treatment
Fritsch, 1984 (1)	Austria	Case series[2]	27	1	1	23Y	M	Not reported
Caputo et al, 1984 (2)	Italy	Case series[2]	2	2	1	34Y	M	Not reported
Traupe et al, 1985 (3)	Germany	Case series[2]	10	1	1	12Y	M	Not reported
Greene et al, 1985 (4)	USA	Case report	1	1	1	20Y	M	Iodoquinol , topical corticosteroids, emollients, antibiotic therapy, antiseptic wet dressings, long tub baths, intermittent antibiotic therapy
Hausser et al, 1989 (5)	Germany	Case report	1	1	1	15Y	F	Not reported
Groves et al, 1995 (6)	Belgium	Case series[2]	2	2	1	11Y	F	Not reported
Braun et al, 1997 (7)	Switzerland	Case report	1	1	1	NR	F	Lactate 12% lotion, PUVA therapy, triamcinolone injections, systemic ibuprofen, tetracosactide, metronidazole, ketoconazole, etretinate, isotretinoin, acitretin
El Shabrawi et al, 2004 (8)	Austria	Case report	1	1	1	29Y	M	Not reported
Lazaridou et al, 2009 (9)	Greece	Case report	1	1	1	14Y	M	Not reported

Reference (author, year)	Country[1]	Study design	Patients (N)	NS patients (N)	Eligible NS patients (N)	Age	Sex	Previous treatment
Renner et al, 2009 (10)	USA	Case series	9	9	3[3]	6Y	M	Moisturizers, topical corticosteroids, antibiotics, allergy control
						2,5Y	M	Moisturizers, topical corticosteroids, antibiotics, allergy control
						7Y	M	Moisturizers, topical corticosteroids, antibiotics, allergy control
Fontao et al, 2011 (11)	Switzerland	Case report	1	1	1	25Y	F	Systemic dapsone, topical corticosteroids, tacrolimus, pimecrolimus
Gallagher et al, 2012 (12)	USA	Case series[2]	2	1	1	At least 4M	F	IVIG
Maatouk et al, 2012 (13)	Lebanon	Case report	1	1	1	16Y	F	Very potent topical corticosteroid (clobetasol propionate)
Small et al, 2016 (14)	USA	Case series	2	2	2	16Y	F	Topical therapies
						10Y	M	Not reported
Yalcin et al, 2016 (15)	Turkey	Case report	1	1	1	20Y	M	Not reported
Roda et al, 2017 (16)	Portugal	Case report	1	1	1	19Y	F	Topical tacrolimus, oral isotretinoin
Eränkö et al, 2018 (17)	Finland	Case series	11	11	3	11Y	NR	Antibiotics
						10Y	NR	Antibiotics
						17Y	NR	Antibiotics
Leung et al, 2018 (18)	Malaysia/ Canada	Case report	1	1	1	8Y	M	Intensive topical emollients, corticosteroids, calcineurin inhibitor

Reference (author, year)	Country[1]	Study design	Patients (N)	NS patients (N)	Eligible NS patients (N)	Age	Sex	Previous treatment
Onnis et al, 2018 (19)	France	Case series[2]	13	1	1	28Y	F	Not reported
Özyurt et al, 2019 (20)	Turkey	Case report	1	1	1	4Y	M	IVIG
Yadav et al, 2019 (21)	India	Case report	1	1	1	23Y	M	Not reported
Aktas et al, 2020 (22)	Turkey	Case report	1	1	1	40Y	F	Topical corticosteroids, retinoids, tacrolimus, acitretin, azathioprine, cyclosporine, IVIG
Andreasen et al, 2020 (23)	Denmark	Case report	1	1	1	43Y	M	Systemic corticosteroids, methotrexate, mycophenolate mofetil, azathioprine, ultraviolet therapy, topical corticosteroids, topical calcineurin inhibitors
Blanchard et al, 2020 (24)	USA	Case report	1	1	1	at least 16Y	M	Topical corticosteroids, oral antibiotics (including cephalexin, azithromycin, clindamycin, doxycycline, amoxicillin-clavulanic acid and linezolid), emollients, oral antihistamines, topical mupirocin, topical clindamycin, tacrolimus 0.03% ointment, pimecrolimus 1% cream, econazole 1% cream, itraconazole, oral ivermectin, acitretin, dapsone, oral prednisone, omalizumab, cyclosporine and narrow-band ultraviolet B

Reference (author, year)	Country[1]	Study design	Patients (N)	NS patients (N)	Eligible NS patients (N)	Age	Sex	Previous treatment
Barbieux et al, 2020 (25)	France	Case series	3	3	3	29Y	F	Emollients, topical corticosteroids, calcineurin inhibitors (6M), anti-histamines, oral antibiotics, alitretinoin (5M), thermal cures
						30Y	M	Emollients, topical and oral corticosteroids, anti-histamines, thermal cures
						20Y	F	Emollients, topical corticosteroids, calcineurin inhibitors, anti-histamines, IVIG (8M), oral antibiotics
Dabas et al, 2020 (26)	India	Case series[2]	6	2	1	13Y	F	Topical emollients, topical and oral corticosteroids, calcineurin inhibitor, growth hormone for stunted growth
Luchsinger et al, 2020 (27)	Switzerland	Case series	4	4	4	21Y	M	Topical corticosteroids and calcineurin inhibitors
						27Y	F	Topical corticosteroids and calcineurin inhibitors, NB–UV-B, adalimumab, alitretinoin
						9Y	M	Topical corticosteroids and calcineurin inhibitors, IVIG, acitretin, alitretinoin
						9Y	M	Topical corticosteroids and calcineurin inhibitors, IVIG
Luchsinger et al, 2020 (28)	Switzerland	Case series[2]	13	1	1	NR	M	Bland emollients, mild to medium-strength topical corticosteroids, calcineurin inhibitors, intravenous immunoglobulins (IVIG) and acitretin
Orlova et al, 2020 (29)	Rusland	Case report	1	1	1	29Y	F	Topical corticosteroids, cetrizine, sodium thiosulfate sol. IV, calcium gluconate sol. 10% 10ml, ebastine, lanolin, nipagin, nipazole, ketotifen, chloropyramine
Steuer et al, 2020 (30)	USA	Case report	1	1	1	32Y	F	Cyclosporine

Reference (author, year)	Country[1]	Study design	Patients (N)	NS patients (N)	Eligible NS patients (N)	Age	Sex	Previous treatment
Süßmuth et al, 2020 (31)	Germany	Case series	2	2	2	12Y	F	Not reported
						8Y	M	Not reported
Volc et al, 2020 (32)	Austria	Case report	1	1	1	15Y	F	Topical and systemic corticosteroids, antibiotics (including oral cephalexin), emollients, acitretin, tacrolimus
Zelieskova et al, 2020 (33)	Slovakia	Case report	1	1	1	12M	M	IVIG
Cicek et al, 2021 (34)	Turkey	Case report	1	1	1	6 M	M	Emollients, antihistamines, antibiotics, topical corticosteroids, topical tacrolimus, and intravenous immunoglobulin
Murase et al, 2021 (35)	Japan	Case series	2	2	2	32Y	F	Repetitive pulse prednisolone therapy 4 times in 7 years for optic nerve inflammation, oral and topical corticosteroids for the skin
						17Y	F	Omalizumab, oral and topical corticosteroids
Zhang et al, 2021 (36)	China	Case report	1	1	1	3Y	M	Topical corticosteroids

Abbreviations: NS= Netherton syndrome; N= number; M= male; F= female; Y=Year; M=Month; NR= not reported; IVIG = intravenous immunoglobulins; USA= United States of America; 1= country of inclusion; 2 = assessed as case report since only one NS patient was included; 3= 5 patients treated with IVIG, but only in 3 patients treatment duration was described and thus 3 patients were eligible for inclusion.

Table S2. Characteristics of included studies evaluating systemic therapy in patients with Netherton syndrome grouped per therapy

Reference (author, year)	Country[1]	Study design (n[3])	Eligible NS patients/ total NS patients (n)	Age	Sex	Concomitant treatment	Treatment	Dosage	Treatment duration	Overall effect[5]	Primary outcome[6,7,8]	Secondary outcomes[6,7,8]	Reported side effects
Hausser et al, 1989 (5)	Germany	Case report (1)	1/1	15Y	F	NR	Acitretin	Initial dose of 35mg/day, decreased to 10 mg every 2nd day after 2 months.	At least 11 months	0	Skin lesions after 4 weeks: +, atopic dermatitis after 8 weeks: -, regression of skin lesions after 11 months: +	After 11 months hair growth : +, hair shaft defects: +, §	Unclear
El Shabrawi et al, 2004 (8)	Austria	Case report (1)	1/1	29Y	M	PUVA	Acitretin	25mg/day	2 weeks	-	Skin condition: -, erythrodermic flare: -	NR	Unclear
Leung et al, 2018 (18)	Malaysia/ Canada	Case report (1)	1/1	8Y	M	Topical moisturizer	Acitretin	0.25mg/kg, reduced to 0.12mg/kg after 6 months for another 6 months	12 months	+	In 2 months skin lesions: +, after 6 months erythema: + and scaling: +	After 2 monthts pruritus: +, after 6 months hair growth and hair condition: +	No reported side effects
Özyurt et al, 2019 (20)	Turkey	Case report (1)	1/1	4Y	M	Topical moisturizers, topical corticosteroids	Acitretin	0.5mg/kg/day	At least 1 year	+	Beneficial effect after 1 year: +, ILC: +	Flare-ups of eczema: +, need to use topical corticosteroids: +	No reported side effects
Yadav et al, 2019 (21)	India	Case report (1)	1/1	23Y	M	Petroleum jelly	Acitretin	25mg/day	2 weeks	-	Within 2 weeks skin condition: -, scaling: -, redness: -	NR	Unclear
Dabas et al, 2020 (26)	India	Case series[2] (6)	1/2	13Y	F	Emollients, antihistamines	Acitretin	0.3mg/kg/day	2 months	0	Improvement: 0	NR	Unclear
Onnis et al, 2018 (19)	France	Case series[2] (13)	1/1	28Y	F	NR	Alitretinoin	Initial dose 10mg/day, increased to 30mg/day after 1 month	6 months	+	Efficacy on prominent signs: +. **VAS** scale before-after treatment: + (6-4); **VAS** erythema: + (8-3), **VAS** palmoplantar keratoderma: + (3-2)	¥	Benign intracranial hypertension, which completely resolved after withdrawal of alitretinoin
Luchsinger et al, 2020 (28)	Switzerland	Case series[2] (13)	1/1	NR	M	IVIG, anithistamines	Alitretinoin	0.5 mg/kg/day	8 months	0	Erythema: 0, other skin findings: 0	NR	Unclear
Greene et al, 1985 (4)	USA	Case report (1)	1/1	20Y	M	Penicillin v potassium for 10 days orally	Isotretinoin	Initial dose of 40mg/day (0.5mg/kg), decreased to 40mg every other day after 3 weeks	15 weeks	0	Initial generalized erythema: -, scaling: - and skin fragility: -. Improvement after 3 weeks: 0	NR	Unclear
Lazaridou et al, 2009 (9)	Greece	Case report (1)	1/1	14Y	M	NR	Isotretinoin	0.2mg/kg	6 months	+	Skin lesions: +	NR	Mild skin dryness, no other reported side effects
Maatouk et al, 2012 (13)	Lebanon	Case report (1)	1/1	16Y	F	NR	Isotretinoin	0.2mg/kg/day	At least 1-2 months	-	Skin condition: -	NR	Unclear

Reference (author, year)	Country[1]	Study design (n[3])	Eligible NS patients/ total NS patients (n)	Age	Sex	Concomitant treatment	Treatment	Dosage	Treatment duration	Overall effect[5]	Primary outcome[6,7,8]	Secondary outcomes[6,7,8]	Reported side effects
Fritsch, 1984 (1)	Austria	Case series[2] (27)	1/1	23Y	M	NR	Etretinate	1mg/kg/day	4 days	-	Erythroderma: -, burning and oozing erosions face and chest: -	NR	Unclear
Caputo et al, 1984 (2)	Italy	Case series[2] (2)	1/2	34Y	M	Topical corticosteroids	Etretinate	75mg/day, then discontinued after 4 days. After re-initiation of treatment 75mg/day in the first 2 months, then 50mg/day for 1 month followed by 50mg every other day	4 days, then discontinued. Approximately 2 years after re-initiation of treatment	0	In the first 4 days atopic dermatitis: -. After re-initiation of treatment ichthyosiform lesions after two months: +	Hair growth after two years: +, Ω	No reported side effects
Traupe et al, 1985 (3)	Germany	Case series[2] (10)	1/1	12Y	M	NR	Etretinate	Initial dose 0.5mg/kg, followed by a maintenance dose of 0.8 mg/kg	At least 22 months	+	ILC after 10 weeks of treatment: +	NR	Unclear
Groves et al, 1995 (6)	Belgium	Case series[2] (2)	1/2	11Y	F	PUVA	Etretinate	25 mg/day	8 years	+	Scaling after 6 weeks: +, intensity of erythema: 0	NR	Mild cheilitis
Orlova et al, 2020 (29)	Russia	Case report (1)	1/1	29Y	F	Unna cream (lanolin), Venofer (iron hydroxide complex III with sucrose), antihistamines, Ketotifen 2 mg/day, Chloropyramine, Omeprazole, Amitriptyline 12.5 mg/day	Systemic prednisolone	90mg per day IV days 1-5; 60 mg per day, days 6-11; 30 mg per day, days 12-15	15 days	+	Skin lesions in 1.5 weeks: +	NR	Unclear
Braun et al, 1997 (7)	Switzerland	Case report (1)	1/1	NR	F	NR	Cyclosporine	Initial dose of 3mg/kg/day , increased to 4 mg/kg /day after 2 months	3 months	0	Skin lesions: 0	NR	Unclear

Reference (author, year)	Country[1]	Study design (n[3])	Eligible NS patients/ total NS patients (n)	Age	Sex	Concomitant treatment	Treatment	Dosage	Treatment duration	Overall effect[5]	Primary outcome[6,7,8]	Secondary outcomes[6,7,8]	Reported side effects
Renner et al, 2009 (10)	USA	Case series (9)	3/9[4]	6Y	M	NR	IVIG	0.4g/kg/month	2 years	+	Inflammation: +	Itchting: +, hair thickness: +, scalp condition: +, number of missed school days: +, number of doctor's visits reduced: +, infection: +, overall quality of life: +, height and weight: +, ¥	Unclear
				2,5Y	M	NR	IVIG	0.4g/kg/month	1 year	+	Inflammation: +	Itchting: +, hair thickness: +, scalp condition: +, number of missed school days: +, number of doctor's visits reduced: +, infection: +, overall quality of life: +, height and weight: +, ¥	Unclear
				7Y	M	NR	IVIG	0.4g/kg/month	6 months	+	Inflammation: +	Itchting: +, hair thickness: +, scalp condition: +, number of missed school days: +, number of doctor's visits reduced: +, infection: +, overall quality of life: +, height and weight: +, ¥	Unclear
Small et al, 2016 (14)	USA	Case series (2)	2/2	16Y	F	NR	IVIG	500 mg/kg monthly	At least 3 months	+	After 3 infusions erythema: +, pustulation: +, scale:+	Pruritus: +, heat tolerance: +, flares: +, secondary infections: +	Unclear
				10Y	M	NR	IVIG	500 mg/kg monthly	At least 3 months	+	After 3 infusions erythema: +, pustulation: +, scale:+	Pruritus: +	Unclear
Eränkö et al, 2018 (17)	Finland	Case series (11)	3/11	11Y	NR	Topical emollients, topical corticosteroids	IVIG	400 mg/kg/month	11 months	+	Skin condition: +, erythema: +.	Pruritus: +, flares: +, tolerance to topical emollients: +, decrease in use of emollient and topical corticosteroids, hair growth: +, hair thickness: +, skin and other infections: +, GER symptoms: 0, ¥	Unclear

Reference (author, year)	Country[1]	Study design (n[3])	Eligible NS patients/ total NS patients (n)	Age	Sex	Concomitant treatment	Treatment	Dosage	Treatment duration	Overall effect[5]	Primary outcome[6,7,8]	Secondary outcomes[6,7,8]	Reported side effects
				10Y	NR	Topical emollients, topical corticosteroids	IVIG	400 mg/kg/month	5 months	+	Skin condition: +, erythema: +.	Pruritus: +, flares:+, tolerance to topical emollients: +, decrease in use of emollient and topical corticosteroids, hair growth: +, hair thickness: +, allergic symptoms: + , GER: +, ¥	Unclear
				17Y	NR	NR	IVIG	400 mg/kg/month	6 months	0	Lack of clear benefits: 0	¥	Unclear
Aktas et al, 2020 (22)	Turkey	Case report (1)	1/1	40Y	F	NR	IVIG	2 g/kg/month	6 months	+	Some improvement: +	NR	Unclear
Blanchard et al, 2020 (24)	USA	Case report (1)	1/1	at least 16Y	M	NR	IVIG	3 monthly infusions at 0.5g/kg	at least 3 months	0	Temporary improvement of truncal rash: +	NR	Unclear
Dabas et al, 2020 (26)	India	Case series[2] (6)	1/2	13Y	F	Emollients, antihistamines	IVIG	Monthly doses of 0.4g/kg/day	6 months	+	Erythema: + and scaling: +	NR	6 months after IVIG treatment: persistent headache caused by thrombosis of left sigmoid and transverse sinus
Zhang et al, 2021 (36)	China	Case report (1)	1/1	3Y	M	Topical corticosteroids	IVIG	500 mg/kg/month	At least 2 months	+	Eruptions: +	NR	Unclear
Gallagher et al, 2012 (12)	USA	Case series[2] (2)	1/1	at least 4M	F	NR	SCIG	NR	47 weeks	+	Ichthyosis: +	Hair thickness: +, pruritus: +, failure to thrive: +, number of illnesses: +, overall quality of life: +, ¥	Local reaction including mild swelling. One SBI (Escherichia coli urinary tract infection) occurred. No serious adverse events were reported
Süßmuth et al, 2020 (31)	Germany	Case series (2)	2/2	8Y	M	NR	SCIG	NR	5 years	+	Clinically more or less beneficial: +	Pruritus: 0	Unclear
Zelieskova et al, 2020 (33)	Slovakia	Case report (1)	1/1	12M	M	NR	SCIG	subcutaneous 20% immunoglobulin substitution (SCIg) 1g every 2 weeks (200mg/kg/month)	At least 1 year	+	Generalized erythroderma: +, ichthyosis: +	Weight gain: +, respiratory morbidity: +	No reported side effects

Reference (author, year)	Country[1]	Study design (n[3])	Eligible NS patients/ total NS patients (n)	Age	Sex	Concomitant treatment	Treatment	Dosage	Treatment duration	Overall effect[5]	Primary outcome[6,7,8]	Secondary outcomes[6,7,8]	Reported side effects
Aktas et al, 2020 (22)	Turkey	Case report (1)	1/1	40Y	F	NR	Dupilumab	Initial dose 600mg , followed by 300mg biweekly	3 months	0	After 6 weeks eczematous lesions: +, by week 10, lesions: -	After 6 weeks pruritus: +, by week 10 pruritus: -	Conjunctivitis, treated with topical antibiotics
Andreasen et al, 2020 (23)	Denmark	Case report (1)	1/1	43Y	M	NR	Dupilumab	Initial dose 600mg , followed by 300mg biweekly	6 months	+	After 4 weeks **EASI**: + (22.6-5.3) , after 6 months NS minimum: +	After 4 weeks **DLQI**: + (19-2), **POEM**: + (15-9), ¥	Unclear
Steuer et al, 2020 (30)	USA	Case report (1)	1/1	32Y	F	Topical 0.1% tacrolimus ointment, tazarotene cream, desonide ointment, emollient moisturizer	Dupilumab	Initial dose 600mg , followed by 300mg biweekly	18 months	+	After 10 months: overall disease severity: +, affected **BSA**: + (reduced by 50%). After 18 months: continued improvement: +	Within 2 months: **NRS** itch: + (9-2). After 10 months functioning: +, travel: +	Unclear
Süßmuth et al, 2020 (31)	Germany	Case series (2)	2/2	12Y	F	Growth hormone, Vitamine D	Dupilumab	Initial dose 600mg , followed by 300mg every 4 weeks, then after 4 months therapy was intensified to 300mg every 2 weeks	12 months	+	After 4 months **NASA**: + (33-11.7), **PGA**: + (4-2), erythema: +, scaling: +. After 12 months: **NASA**: + (33-11.7), **PGA**: + (4-2)	**NRS** pruritus 4 months: + (8-3). After 12 months **NRS** pruritus: + (8-2). Overall frequency of cutaneous infections: +, ¥	Bacterial superinfection which led to temprary increase of NASA after 10 months, treated with topical antiseptics
				8Y	M	SCIG	Dupilumab	300 mg every 4 weeks	10 months	+	**NASA** after 10 months: + (50.5-18). **PGA** 10 months: + (4-2), erythema: +, scaling: +	**NRS** pruritus 10 months: + (8-3)	No reported side effects
Murase et al, 2021 (35)	Japan	Case series (2)	2/2	32Y	F	NR	Dupilumab	Initial dose of 600mg, followed by 300mg every 2 weeks	6 months	+	After 6 months **EASI**: + (61.8-12.9), **IGA**: + (4-2), **CIS**: + (49.2-21)	1 day after injection: pruritus +, After 6 months: **VAS** itching: + (7-3), hair growth: +, hair strength: +, **HAP**: +, **HSD**: +, ¥	No reported side effects
				17Y	F	NR	Dupilumab	Initial dose of 600mg, followed by 300mg every 2 weeks	6 months	+	After 6 months **EASI**: + (35.6-12.4), **IGA**: + (4-2), **CIS**: + (39-30.5)	1 day after injection: pruritus +, After 6 months **VAS** itching: + (5-3), hair growth: +, hair strength: +, **HAP**: 0, **HSD**: 0, ¥	No reported side effects

Reference (author, year)	Country[1]	Study design (n[3])	Eligible NS patients/ total NS patients (n)	Age	Sex	Concomitant treatment	Treatment	Dosage	Treatment duration	Overall effect[5]	Primary outcome[6,7,8]	Secondary outcomes[6,7,8]	Reported side effects
Barbieux et al, 2020 (25)	France	Case series (3)	3/3	29Y	F	Emollients, topical corticosteroids, calcineurin inhibitors (6M), anti-histamines, oral antibiotics, alitretinoin (5M), thermal cures	Ixekizumab	Starting dose of 2x80mg followed by bi-monthly subcutaneous injections of 80mg during 12 weeks and then monthly injections of 80mg during 12 weeks	6 months	+	In the first 12 weeks: cutaneous improvement: +, oozing: + , scaling: +, **IASI-S**: +. After 6 months **IASI-E**: 0, **IASI-S**: +	In the first 12 weeks: flares: +, pruritus: +, reduction in use of topical corticosteroids: +, **DLQI**: +. At 6 months **DLQI**: +, pruritus: +, §¥	Unclear
				30Y	M	Emollients, topical and oral corticosteroids, anti-histamines, thermal cures	Ixekizumab	Starting dose of 2x80mg followed by bi-monthly subcutaneous injections of 80mg during 12 weeks and then monthly injections of 80mg during 12 weeks	6 months	0	In the first 12 weeks: cutaneous improvement: +, oozing: + , scaling: +, **IASI-S**: +. After 6 months **IASI-E**: 0, **IASI-S**: 0	In the first 12 weeks: flares: +, pruritus: +, reduction in use of topical corticosteroids: +, **DLQI**: +. At 6 months **DLQI**: 0, pruritus: 0, §¥	Unclear
				20Y	F	Emollients, topical corticosteroids, calcineurin inhibitors, anti-histamines, IVIG (8M), oral antibiotics	Ixekizumab	Starting dose of 2x80mg followed by bi-monthly subcutaneous injections of 80mg during 12 weeks and then monthly injections of 80mg during 12 weeks	6 months	+	In the first 12 weeks: cutaneous improvement: +, oozing: + , scaling: +, **IASI-S**: +. After 6 months **IASI-E**: 0, **IASI-S**:+	In the first 12 weeks: flares: +, pruritus: +, reduction in use of topical corticosteroids: +, **DLQI**: +. At 6 months **DLQI**: +, pruritus: +, §¥	Unclear
Blanchard et al, 2020 (24)	USA	Case report (1)	1/1	at least 16Y	M	Moisturizer and tretinoin 0.025% cream	Secukinumab	300mg weekly in the first month, followed by 300mg monthly	Almost 3 years	+	After 4 weeks facial and truncal rash: +. After 3 years facial erythema: +	Flare: +	Unclear

Reference (author, year)	Country[1]	Study design (n[3])	Eligible NS patients/ total NS patients (n)	Age	Sex	Concomitant treatment	Treatment	Dosage	Treatment duration	Overall effect[5]	Primary outcome[6,7,8]	Secondary outcomes[6,7,8]	Reported side effects
Luchsinger et al, 2020 (27)	Switzerland	Case series (4)	4/4	21Y	M	Topical corticosteroids	Secukinumab	Weekly from baseline until 4 weeks, then monthly dose (Dosage mentioned as mg/kg based on weight categories, weight and total dosage per patient not reported)	3 months	+	Cutaneous improvement after 2 doses: +. After 3 months **IASI**, **IASI-S**, **IASI-E**: +.	After 3 months **DLQI**: +, **5-D itch scale**: +, use of topical steroids: +, night sleep: +	Onychomycosis due to candida albicans occurred in 2 patients, common viral warts occurred in 1 patients. Both children developed acute pruritic palmoplantar eczematous reaction, refractory to topical corticosteroids
				27Y	F	Topical corticosteroids	Secukinumab	Weekly from baseline until 4 weeks, then monthly dose	12 months	+	Cutaneous improvement after 2 doses: +. After 3 and 6 months **IASI**, **IASI-S**, **IASI-E**: +.	After 3 and 6 months **DLQI**: +, **5-D itch scale**: +, use of topical steroids: +, night sleep: +	
				9Y	M	Topical corticosteroids	Secukinumab	Weekly from baseline until 4 weeks, then monthly dose	8 months	+	Cutaneous improvement after 2 doses: +. After 3 and 6 months **IASI**, **IASI-S**, **IASI-E**: +.	After 3 and 6 months **DLQI**: +, **5-D itch scale**: +, chronic blepharitis: +, recurrent otitis externa: +, use of topical steroids: +, night sleep: +, continued weight gain: +, growth rate: +	
				9Y	M	Topical corticosteroids	Secukinumab	Weekly from baseline until 4 weeks, then monthly dose	7 months	+	Cutaneous improvement after 2 doses: +. After 3 and 6 months **IASI**, **IASI-S**, **IASI-E**: +.	After 3 and 6 months **DLQI**: +, **5-D itch scale**: +, chronic blepharitis: +, skin infections: +, use of topical steroids: +, night sleep: +, use of topical corticosteroids, continued weight gain: +, growth rate: +	
Fontao et al, 2011 (11)	Switzerland	Case report (1)	1/1	25Y	F	NR	Infliximab	5mg/kg in weeks 0-2-6, then continued every 4 weeks	2 years	+	Clinical improvement after second infusion: +, after 12 infusions inflammatory skin lesions: +, xerosis: 0 , ichthyosis: 0	Number of infections: +, §¥	No reported side effects
Roda et al, 2017 (16)	Portugal	Case report (1)	1/1	19Y	F	NR	Infliximab	5mg/kg in weeks 0-2-6, then continued every 8 weeks	(at least) 22 weeks	+	Clinical improvement after the second infusion: +, inflammatory lesions: +, desquamation: +. At week 22 inflammatory lesions: +	Pruritus: +, hair strength and hair length: +	Unclear

Reference (author, year)	Country[1]	Study design (n[3])	Eligible NS patients/ total NS patients (n)	Age	Sex	Concomitant treatment	Treatment	Dosage	Treatment duration	Overall effect[5]	Primary outcome[6,7,8]	Secondary outcomes[6,7,8]	Reported side effects
Cicek et al, 2021 (34)	Turkey	Case report (1)	1/1	6M	M	NR	Infliximab	5mg/kg given in weeks 0-2-6, then continued every 4 weeks	1 year	+	After third infusion: skin and scalp: +, after 1 year of treatment, skin and scalp rash: +	NR	No reported side effects
Yalcin et al, 2016 (15)	Turkey	Case report (1)	1/1	20Y	M	H1 (Chlorphenoxamine, 10 mg)/H2 (Rantidine, 50 mg) blockers (intramuscular) and pulse prednisolone 250 mg once in a week for 4 weeks	Omalizumab	400mg/kg	4 months	+	Clinical improvement: +, histologically reepithelialization below the necrotic epidermis after 2 weeks: +	Use of steroid treatment: +, ¥	Unclear
Blanchard et al, 2020 (24)	USA	Case report (1)	1/1	at least 16Y	M	NR	Adalimumab	40 mg every other week starting 1 week after an 80mg loading dose	At least 6 months	0	Intitial response of the facial rash: +, efficacy within 6 months-	NR	Unclear
Volc et al, 2020 (32)	Austria	Case report (1)	1/1	15Y	F	3-monthly injections of medroxyprogesterone acetate	Ustekinumab	45 mg (0.75mg/kg)	At least 1 year	+	After 4 weeks skin symptoms: +, eczematous areas and psoriasiform lesions: +, After 1 year well-doing: +	Signs of Cushing syndrome: +.	Unclear

Abbreviations: NS= Netherton syndrome; N= number; M= male; F= female; Y=Year; M=Month; NR= not reported; USA= United States of America; NASA = Netherton Area Severity Assessment; PGA = Physician Global Assessment; VAS = Visual Analogue Scale; NRS= Numerical Rating Scale; IGA= Investigator Global Assessment; IASI = Ichthyosis Area and Severity Index; IASI-S = Ichthyosis Area and Severity Index - Scaling; IASI-E = Ichthyosis Area and Severity Index - Erythema; EASI = Eczema Area and Severity Index; BSA= Body Surface Area; DLQI = Dermatology Life Quality Index; POEM = Patient Oriented Eczema Measure; CIS= Clinical Ichthyosis Score; HAP= Hair area percentage; HSD= Hair shaft diameter; ILC = Ichthyosis Linearis Circumflexa; IVIG = intravenous immunoglobulins; SCIG= subcutaneous immunoglobulins; SBI= serious bacterial infection; PUVA = psoralen-UVA- therapy; GER = gastro-esophageal reflux; mg = milligrams; g= grams, kg = kilograms; 1= country of patient inclusion; 2 = assessed as case report since only one NS patient was included; 3= total number of patients included in the study; 4= 5 patients treated with IVIG, but only in 3 patients treatment duration was described and thus 3 patients were eligible for inclusion; 5= '+' indicates improvement of the skin condition, '-' indicates worsening of the skin condition, '0' indicates no change; or temporary improvement; or a combination of worsening and improvement; or temporary worsening of the skin condition; 6= '+'indicates improvement, '0' indicates no change, and '-' indicates worsening ; 7= if absolute numbers were mentioned in the study these are presented as (before-after); 8= measurement instruments are shown in bold; §= skin biopsy measurements taken prior to and after treatment ; ¥= blood measurements taken prior to and after treatment; Ω= urinary amino acids levels measurements taken prior to and after treatment.

References

1. Fritsch P. Austrian Experience with Etretinate. In: Cunliffe WJ, Miller AJ, editor. Retinoid therapy : a review of clinical and laboratory research: the proceedings of an international conference held in London, 16-18 May 1983. Lancaster; Boston: MTP Press; 1984. p. 45-54.

2. Caputo R, Vanotti P, Bertani E. Netherton's syndrome in two adult brothers. Arch Dermatol. 1984;120(2):220-2.

3. Traupe H, Happle R. Etretinate therapy in children with severe keratinization defects. Eur J Pediatr. 1985;143(3):166-9. DOI: 10.1007/BF00442128.

4. Greene SL, Muller SA. Netherton's syndrome. Report of a case and review of the literature. J Am Acad Dermatol. 1985;13(2 Pt 2):329-37.

5. Hausser I, Anton-Lamprecht I, Hartschuh W, Petzoldt D. Netherton's syndrome: ultrastructure of the active lesion under retinoid therapy. Arch Dermatol Res. 1989;281(3):165-72. DOI: 10.1007/BF00456387.

6. Groves S. DB, Bonardeaux Ch., De la Brassinne M. Netherton's syndrome in two sisters. A ten year experience of therapy with retinoids. Journal of the European Academy of Dermatology and Venereology. 1995.

7. Braun RP, Ramelet AA. Failure of cyclosporine in Netherton's syndrome. Dermatology. 1997;195(1):75. DOI: 10.1159/000245696.

8. El Shabrawi-Caelen L, Smolle J, Metze D, Ginter-Hanselmayer G, Raghunath M, Traupe H, et al. Generalized exfoliative erythroderma since birth. Netherton syndrome. Arch Dermatol. 2004;140(10):1275-80. DOI: 10.1001/archderm.140.10.1275-a.

9. Lazaridou E, Apalla Z, Patsatsi A, Trigoni A, Ioannides D. Netherton's syndrome: successful treatment with isotretinoin. J Eur Acad Dermatol Venereol. 2009;23(2):210-2. DOI: 10.1111/j.1468-3083.2008.02795.x.

10. Renner ED, Hartl D, Rylaarsdam S, Young ML, Monaco-Shawver L, Kleiner G, et al. Comel-Netherton syndrome defined as primary immunodeficiency. J Allergy Clin Immunol. 2009;124(3):536-43. DOI: 10.1016/j.jaci.2009.06.009.

11. Fontao L, Laffitte E, Briot A, Kaya G, Roux-Lombard P, Fraitag S, et al. Infliximab infusions for Netherton syndrome: sustained clinical improvement correlates with a reduction of thymic stromal lymphopoietin levels in the skin. J Invest Dermatol. 2011;131(9):1947-50. DOI: 10.1038/jid.2011.124.

12. Gallagher JL, Patel NC. Subcutaneous immunoglobulin replacement therapy with Hizentra(R) is safe and effective in two infants. J Clin Immunol. 2012;32(3):474-6. DOI: 10.1007/s10875-011-9645-0.

13. Maatouk I, Moutran R, Tomb R. Narrowband ultraviolet B phototherapy associated with improvement in Netherton syndrome. Clin Exp Dermatol. 2012;37(4):364-6. DOI: 10.1111/j.1365-2230.2011.04231.x.

14. Small AM, Cordoro KM. Netherton Syndrome Mimicking Pustular Psoriasis: Clinical Implications and Response to Intravenous Immunoglobulin. Pediatr Dermatol. 2016;33(3):e222-3. DOI: 10.1111/pde.12856.

15. Yalcin AD. A case of netherton syndrome: successful treatment with omalizumab and pulse prednisolone and its effects on cytokines and immunoglobulin levels. Immunopharmacol Immunotoxicol. 2016;38(2):162-6. DOI: 10.3109/08923973.2015.1115518.

16. Roda A, Mendonca-Sanches M, Travassos AR, Soares-de-Almeida L, Metze D. Infliximab therapy for Netherton syndrome: A case report. JAAD Case Rep. 2017;3(6):550-2. DOI: 10.1016/j.jdcr.2017.07.019.

17. Eranko E, Ilander M, Tuomiranta M, Makitie A, Lassila T, Kreutzman A, et al. Immune cell phenotype and functional defects in Netherton syndrome. Orphanet J Rare Dis. 2018;13(1):213. DOI: 10.1186/s13023-018-0956-6.

18. Leung AKC, Barankin B, Leong KF. An 8-Year-Old Child with Delayed Diagnosis of Netherton Syndrome. Case Rep Pediatr. 2018;2018:9434916. DOI: 10.1155/2018/9434916.

19. Onnis G, Chiaverini C, Hickman G, Dreyfus I, Fischer J, Bourrat E, et al. Alitretinoin reduces erythema in inherited ichthyosis. Orphanet J Rare Dis. 2018;13(1):46. DOI: 10.1186/s13023-018-0783-9.

20. Ozyurt K, Atasoy M, Ertas R, Ulas Y, Akkus MR, Kiraz A, et al. Netherton syndrome previously misdiagnosed as hyper IgE syndrome caused by a probable mutation in SPINK5 C. Turk J Pediatr. 2019;61(4):604-7. DOI: 10.24953/turkjped.2019.04.020.

21. Yadav N, Madke B, Kar S, Gangane N. Netherton syndrome: an atypical presentation. Cutis. 2019;103(4):E27-E9.

22. Aktas M, Salman A, Apti Sengun O, Comert Ozer E, Hosgoren Tekin S, Akin Cakici O, et al. Netherton syndrome: Temporary response to dupilumab. Pediatr Dermatol. 2020;37(6):1210-1. DOI: 10.1111/pde.14362.

23. Andreasen TH, Karstensen HG, Duno M, Lei U, Zachariae C, Thyssen JP. Successful treatment with dupilumab of an adult with Netherton syndrome. Clin Exp Dermatol. 2020;45(7):915-7. DOI: 10.1111/ced.14317.

24. Blanchard SK, Prose NS. Successful use of secukinumab in Netherton syndrome. JAAD Case Rep. 2020;6(6):577-8. DOI: 10.1016/j.jdcr.2020.04.025.

25. Barbieux C, Bonnet des Claustres M, de la Brassinne M, Bricteux G, Bagot M, Bourrat E, et al. Duality of Netherton syndrome manifestations and response to ixekizumab. J Am Acad Dermatol. 2021;84(5):1476-80. DOI: 10.1016/j.jaad.2020.07.054.

26. Dabas G, Mahajan R, De D, Handa S, Kumar R, Dayal D, et al. Managing syndromic congenital ichthyosis at a tertiary care institute-Genotype-phenotype correlations, and novel treatments. Dermatol Ther. 2020;33(6):e13816. DOI: 10.1111/dth.13816.

27. Luchsinger I, Knopfel N, Theiler M, Bonnet des Claustres M, Barbieux C, Schwieger-Briel A, et al. Secukinumab Therapy for Netherton Syndrome. JAMA Dermatol. 2020;156(8):907-11. DOI: 10.1001/jamadermatol.2020.1019.

28. Luchsinger I, Vogler T, Schwieger-Briel A, Knopfel N, Walchli R, Weibel L, et al. Safe and effective use of alitretinoin in children with recalcitrant hand eczema and other dermatoses - a retrospective analysis. J Eur Acad Dermatol Venereol. 2020;34(5):1037-42. DOI: 10.1111/jdv.16088.

29. Orlova E, Smirnova L, Grabovskaya O, Kayumova L. Netherton syndrome in combination with iron-deficiency anemia. Journal of Global Pharma Technology. 2020;12(1):12-21.

30. Steuer AB, Cohen DE. Treatment of Netherton Syndrome With Dupilumab. JAMA Dermatol. 2020;156(3):350-1. DOI: 10.1001/jamadermatol.2019.4608.

31. Sussmuth K, Traupe H, Loser K, Stander S, Kessel C, Wittkowski H, et al. Response to dupilumab in two children with Netherton syndrome: Improvement of pruritus and scaling. J Eur Acad Dermatol Venereol. 2021;35(2):e152-e5. DOI: 10.1111/jdv.16883.

32. Volc S, Maier L, Gritsch A, Aichelburg MC, Volc-Platzer B. Successful treatment of Netherton syndrome with ustekinumab in a 15-year-old girl. Br J Dermatol. 2020;183(1):165-7. DOI: 10.1111/bjd.18892.

33. Zelieskova M, Banovcin P, Kozar M, Kozarova A, Nudzajova Z, Jesenak M. A novel SPINK5 mutation and successful subcutaneous immunoglobulin replacement therapy in a child with Netherton syndrome. Pediatr Dermatol. 2020;37(6):1202-4. DOI: 10.1111/pde.14318.

34. Cicek F, Cekic S, Kilic SS. Infliximab therapy in an infant with Netherton syndrome. Pediatr Dermatol. 2021;38(3):714-6. DOI: 10.1111/pde.14590.

35. Murase C, Takeichi T, Taki T, Yoshikawa T, Suzuki A, Ogi T, et al. Successful dupilumab treatment for ichthyotic and atopic features of Netherton syndrome. J Dermatol Sci. 2021;102(2):126-9. DOI: 10.1016/j.jdermsci.2021.03.003.

36. Zhang Z, Pan C, Wei R, Li H, Yang Y, Chen J, et al. Netherton syndrome caused by compound heterozygous mutation, c.80A>G mutation in SPINK5 and large-sized genomic deletion mutation, and successful treatment of intravenous immunoglobulin. Mol Genet Genomic Med. 2021;9(3):e1600. DOI: 10.1002/mgg3.1600.
